# Clinical courses of acute kidney injury in hospitalized patients: a multistate analysis

**DOI:** 10.1038/s41598-023-45006-5

**Published:** 2023-10-18

**Authors:** Esra Adiyeke, Yuanfang Ren, Ziyuan Guan, Matthew M. Ruppert, Parisa Rashidi, Azra Bihorac, Tezcan Ozrazgat-Baslanti

**Affiliations:** 1 Intelligent Clinical Care Center, University of Florida, Gainesville, FL USA; 2 Department of Medicine. Division of Nephrology, Hypertension, and Renal Transplantation, University of Florida, PO Box 100224, Gainesville, FL 32610-0224 USA; 3 Department of Biomedical Engineering, University of Florida, Gainesville, FL USA

**Keywords:** Machine learning, Statistical methods, Acute kidney injury

## Abstract

Persistence of acute kidney injury (AKI) or insufficient recovery of renal function was associated with reduced long-term survival and life quality. We quantified AKI trajectories and describe transitions through progression and recovery among hospitalized patients. 245,663 encounters from 128,271 patients admitted to UF Health between 2012 and 2019 were retrospectively categorized according to the worst AKI stage experienced within 24-h periods. Multistate models were fit for describing characteristics influencing transitions towards progressed or regressed AKI, discharge, and death. Effects of age, sex, race, admission comorbidities, and prolonged intensive care unit stay (ICU) on transition rates were examined via Cox proportional hazards models. About 20% of encounters had AKI; where 66% of those with AKI had Stage 1 as their worst AKI severity during hospitalization, 18% had Stage 2, and 16% had Stage 3 AKI (12% with kidney replacement therapy (KRT) and 4% without KRT). At 3 days following Stage 1 AKI, 71.1% (70.5–71.6%) were either resolved to No AKI or discharged, while recovery proportion was 38% (37.4–38.6%) and discharge proportion was 7.1% (6.9–7.3%) following AKI Stage 2. At 14 days following Stage 1 AKI, patients with additional frail conditions stay had lower transition proportion towards No AKI or discharge states. Multistate modeling framework is a facilitating mechanism for understanding AKI clinical course and examining characteristics influencing disease process and transition rates.

## Background and significance

Acute kidney injury (AKI) occurs in almost 25% of patients admitted to hospitals and up to 60% of patients receiving critical care^1–3^. Persistence of AKI or insufficient recovery of renal function exacerbates risk for adverse health conditions and worsens long-term survival in addition to patients’ well-being^4,5^. To optimize and tailor clinical actions and their timely delivery, it is imperative to understand clinical course of AKI in terms of severity and recovery during hospitalization.

Conventional survival analysis methods have been utilized to describe AKI trajectories and associated outcomes^4,6,7^. These models have the capacity to deal with time-to-event type data and censored subjects, where a subject being censored refers to failing to experience the study’s event of interest or being dropped out of the study by the end of the observation period or follow-up time^8,9^. Despite their significant merits, traditional survival analysis methods, such as Kaplan–Meier methods, have certain limitations. For instance, censoring action used in these models could be considered uninformative since in real-world scenarios patients are subject to several competing risks. Competing risks models could deal with aforementioned structures; however, both approaches treat all states as *absorbing* and therefore lack inclusion of patients’ history. For analyses that involve patient histories with several events of interest occurrence, multistate models could be used to characterize the competing risks. Applications of multistate models could be found for various care levels^10,11^ and patient groups such as kidney disease^12^, diabetic^13^ , surgical^14^ , cancer^15^ , COVID-19^16^, and geriatric^17^ cohorts.

Multistate models are specifically beneficial in analyzing temporal changes and present an alternative approach with considerable potential in research studies with longitudinal nature; however, multistate models require precise and detailed records of transitions between the identified states. Consequently, we retrospectively performed multistate-based analyses on a large cohort of subjects with the following objectives: (1) to understand the clinical course of AKI among hospitalized patients by estimating the probability of being in a specific clinical state at a certain time after entering each one of the AKI stages, and (2) to investigate the effects of age, sex, race, comorbidities, and prolonged ICU stay on transition rates via Cox proportional hazards regression models.

## Methods

### Study design

The study was designed and approved by the Institutional Review Board of the University of Florida and the University of Florida Privacy Office as an exempt study with waiver of informed consent (IRB 201901123). The University of Florida Health (UFH) Integrated Data Repository acted as Honest Broker. We performed all methods in accordance with the relevant guidelines and regulations. A single-center, longitudinal dataset was curated from the electronic health records of 156,699 adult patients admitted to UFH between January 1, 2012, and August 22, 2019. We excluded patients with end stage kidney disease (ESKD) encounters with no serum creatinine measurement to determine AKI status during hospitalization and within 48 h of hospital admission, and encounters discharged (alive or dead) within 24 h of admission. Our final cohort included 245,663 hospital encounters from 128,271 patients (Supplementary Fig. S1). This study followed Strengthening the Reporting of Observational Studies in Epidemiology (STROBE) recommendations^18^.

### Assessment of kidney function and study outcomes

In identifying and staging the AKI, we used a validated computable phenotyping algorithm^19^ that relies on Kidney Disease: Improving Global Outcomes (KDIGO) serum creatinine criteria^20–22^. Reference creatinine was determined using preadmission records^23^ or estimated using the Chronic Kidney Disease Epidemiology Collaboration (CKD-EPI) Study equation refit without race multiplier, as per recommendations, with a baseline estimated glomerular filtration rate assigned to 75 ml/min/per 1.73 m^2^^22,24–26^. We identified primary clinical outcomes as No AKI, AKI Stage 1, AKI Stage 2, AKI Stage 3 without kidney replacement therapy (KRT), AKI Stage 3 with KRT, hospital death, and discharge (Supplementary Fig. S2). Details regarding assumptions and the phenotyping algorithm pipeline can be found in Ozrazgat-Baslanti et al.^19^.

### Multistate analyses

Multistate models allow intermediate events to simultaneously change the risk of reaching a terminal state^13^. In defining and fitting a multistate model, state set and transition set need to be identified. In this context, state set represents temporal status of a patient whereas transition set defines possible movements between states. A state is considered absorbing (or terminal) if leaving that particular state is impossible, and a state is considered transient if transitioning to another state is possible. Basically, state set of a multistate model is a collection of initial state(s), transient state(s), and terminal state(s). An initial state is the time point of the subject’s entry into the model, and returning to an initial state is not possible once it was left. Apart from absorbing and initial states, remaining transient states could be visited several times. The collection of states and transitions presents the framework for designating a statistical model for hazard function for each of the transitions identified.

Multistate models assist in quantifying separate transition intensities for switching from one particular state to another state and in quantifying the present proportion of the patients occupying a specified state at a given time point. Therefore, these models allow estimating the probability of a clinical event occurring after an entrance to a particular state over an extended time. We refer the reader for relevant background of the non-parametric or semi-parametric models to Andersen et al.^27^, Thernau et al.^28^, and Geskus et al.^29^.

We developed two separate multistate models by using a large dataset that assembles both time-varying and static information of the patients. We identified 8 mutually exclusive states based on patients’ clinical condition at each time point. These states are enumerated and listed as follows: (0) Admission, (1) No AKI, (2) AKI Stage 1, (3) AKI Stage 2, (4) AKI Stage 3 without KRT, (5) AKI Stage 3 with KRT, (6) Death, and (7) Discharge (Supplementary Fig. S2). States were discretely determined by considering the worst AKI condition a subject experienced within 24-h time periods.

**Figure 1:**
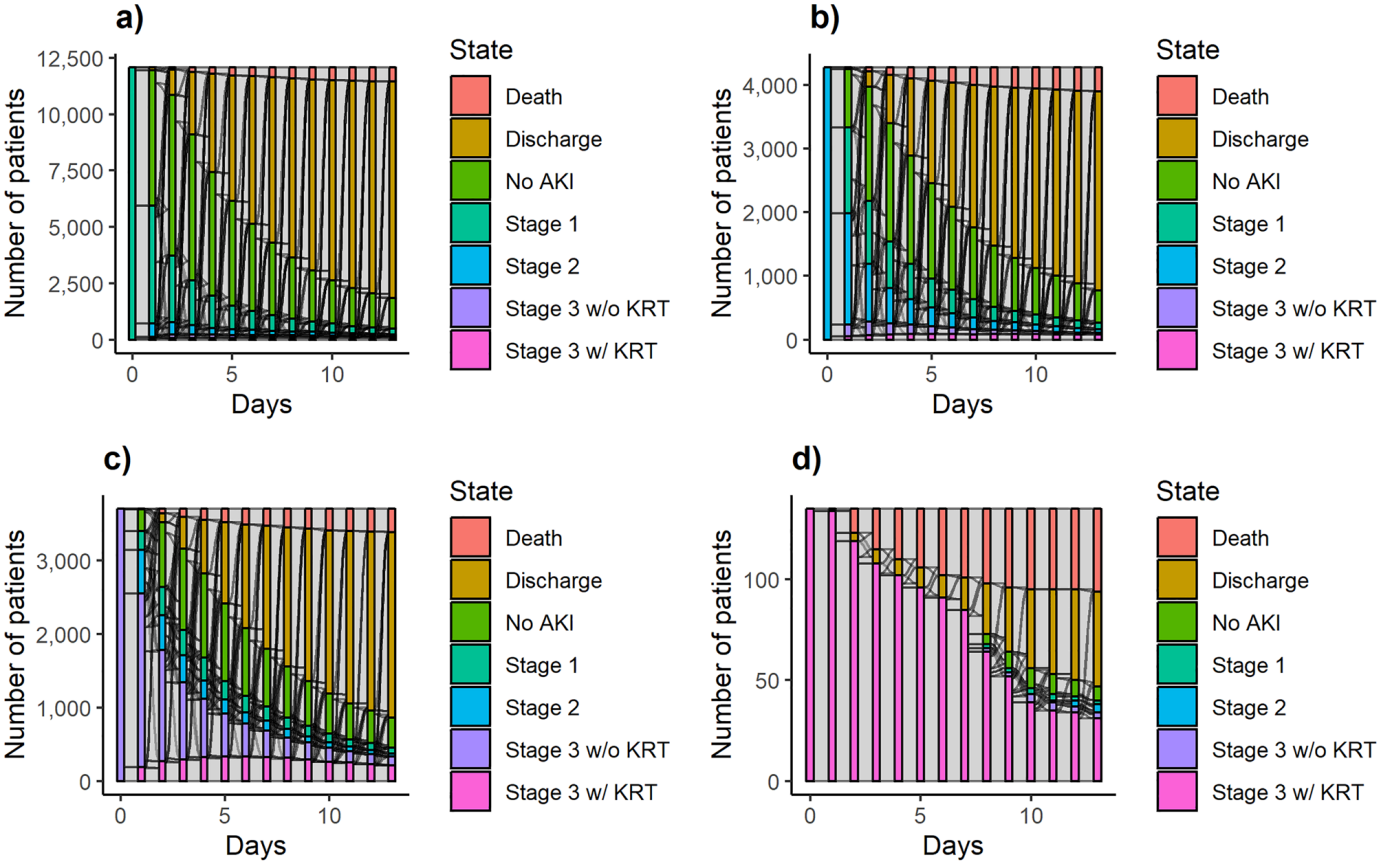
Number of patients transitioning on consecutive days shown for 14 days of hospitalization for AKI Stage 1 (**a**), AKI Stage 2 (**b**), AKI Stage 3 without KRT (**c**), and AKI Stage 3 with KRT (**d**) patients. The horizontal axis of the figures in the panels represent the days in hospital and the vertical axis displays the number of patients.

We first quantified the transition probabilities using an Aalen-Johansen estimation-based non-parametric multistate model where the variable effects were ignored in estimating transition probabilities from one state to another. Following that, in describing the variable effects on the hazards, we fit a Cox model in the multistate semi-parametric framework. This approach aids in specifying distinct variable effects for distinct transitions in the terminal states either with or without inclusion of intermediate events. We included age, sex, race, Charlson comorbidity index (CCI), and prolonged ICU stay (e.g., ICU stay longer than 48 h) as the variables^30^. We presented the clinical course of the AKI patients via alluvial plots where we stratified the patients by considering their movements between the specified states throughout their hospitalization (Fig. 1)^31^.

We calculated instantaneous hazard rates for particular events from predetermined states without considering intermediate events. Specifically, the instantaneous hazard rates from No AKI, AKI Stage 1, AKI Stage 2, and AKI Stage 3 states to these predetermined states including death state were calculated. For all analyses performed, we considered time 0 as the entrance moment into a certain state. Patients were censored at hospital discharge, death, or the end of the 14-day of observation period, whichever came first. Patient characteristics were presented in terms of their means and standard deviations (SD), medians with interquartile ranges (IQR), or frequencies with percentages, as appropriate. Kruskall-Wallis test or chi-square tests were performed to compare the data between groups, where appropriate. Threshold was set to 0.05 for a *p*-value to indicate statistical significance. All data processing and analyses were performed using Python 3.8 and R 4.1.2. We conducted modeling and statistical analyses using mstate and survival packages^32–35^.

## Results

### Clinical characteristics of patients

Subjects were categorized with respect to the worst AKI severity outcome during their hospitalization. Cohort characteristics and their statistical comparisons were reported in Table 1. Average age for No AKI patients was 55 and was significantly lower than AKI groups (Table 1). Female patients were the majority for No AKI and Stage 2 AKI, whereas male patients were the majority for remaining patient groups. Percentage of CCI ≥ 3 was the lowest for No AKI group. Hospital length of stay was significantly lower for No AKI cohort. Similarly, time period with mechanical ventilation and ICU length of stay was lower for No AKI group. Detailed cohort characteristics and outcomes were reported in Supplementary Table S1.

**Table 1:** Detailed cohort characteristics and outcomes stratified by worst AKI severity.

Features	No AKI	AKI Stage 1	AKI Stage 2	AKI Stage 3 without KRT	AKI Stage 3 with KRT
Number of encounters, n	195,581	33,260	8,779	6,004	2,039
Age in years, mean (SD)	55 (18)	**61 (17)**	**61 (17)**	**60 (16)**	**59 (15)**
Female, n (%)	103,009 (53)	**16,451 (49)**	4,580 (52)	**2,855 (48)**	**802 (39)**
Hispanic ethnicity, n (%)	8,257 (4)	**1,216 (4)**	**283 (3)**	209 (3)	73 (4)
African American, n (%)	38,959 (20)	**7,434 (22)**	**1,882 (21)**	**1,543 (26)**	423 (21)
CKD, n (%)	40,573 (21)	**12,988 (39)**	**2,909 (33)**	**3,002 (50)**	**1,194 (59)**
Charlson comorbidity index, median (IQR)	1 (0, 3)	**2 (1, 4)**	**2 (1, 4)**	**3 (1, 5)**	**3 (2, 5)**
Reference creatinine, mean (SD)	0.84 (0.32)	**0.97 (0.47)**	**0.85 (0.30)**	**1.53 (1.61)**	**1.63 (1.58)**
Mechanical ventilation days, median (IQR)	2 (2, 4)	**3 (2, 7)**	**4 (2, 9)**	**5 (3, 10)**	**9 (4, 18)**
Length of stay (days), median (IQR)	3 (2, 6)	**7 (4, 12)**	**8 (4, 16)**	**8 (5, 16)**	**21 (10, 35)**
ICU length of stay (days), median (IQR)	3 (2, 6)	**5 (3, 10)**	**7 (4, 13)**	**6 (4, 13)**	**15 (7, 28)**
ICU length of stay ≥ 48 h, n (%)	29,987 (15)	**126,25 (38)**	**4,385 (50)**	**3,133 (52)**	**1,803 (88)**
Hospital mortality, n (%)	2,361 (1)	**1,593 (5)**	**993 (11)**	**1,097 (18)**	**920 (45)**

AKI, acute kidney injury; CKD, chronic kidney disease; ICU, intensive care unit; IQR, interquartile range; KRT, kidney replacement therapy; SD, standard deviation.

Bold values in Table 1 indicate a Bonferroni corrected *p*-value ≤ 0.05 compared to no AKI group.

### AKI trajectory analyses

Daily transitions were demonstrated in Fig. 1 for each AKI stage. Patients admitted with AKI Stage 1 had the highest proportion for early resolution or discharge and the lowest percentage for AKI progression (Fig. 1a). Patients admitted with worse AKI severity had almost consistently lower proportions for resolution and discharge compared to AKI Stage 1 group. Similarly, patients with more severe AKI groups on their early days of hospitalization had higher percentages for maintaining their initial AKI stage (Fig. 1b–d).

Within 24 h following the admission, 8.22% of the patients had AKI, and a majority of those AKI patients had Stage 1 AKI (Fig. 1, Table 2). From 7 days after admission, 2.09% (95% Confidence Interval (CI) 2.04–2.15%) of all patients had Stage 1 AKI, whereas 1.43% (95% CI 1.35–1.50%) experienced Stage 2 or more severe AKI. At that point, 66.39% (95% CI 66.22–66.56%) of the cohort were discharged, and the probability for terminal state of death was 1.19% (95% CI 1.16–1.23%). On day 7 following AKI Stage 2, proportion of progression to higher stages of AKI (5.67% [95% CI 5.35–6.02%]) was higher than the proportion of progression to higher stages from AKI Stage 1 (2.90% [95% CI 2.74–3.08%]). At that time point, resolved or discharged percentage of the patients with an initial status of AKI Stage 1 was higher (91.85% [95% CI 91.46–92.23%]) than cohorts with AKI Stage 2, 3 without KRT, and 3 with KRT. Among AKI patients without KRT requirement, Stage 3 patients had the highest percentage for persisting condition (12.24% [95% CI 11.60–12.91%]).

**Table 2:** Multistate models based estimated proportions of patients in each clinical state over time.

Patient Characteristics and Outcomes	No AKI, % (95% CI)	AKI Stage 1, % (95% CI)	AKI Stage 2, % (95% CI)	AKI Stage 3 without KRT, % (95% CI)	AKI Stage 3 with KRT, % (95% CI)	Death, % (95% CI)	Discharge, % (95% CI)
Days since admission							
Day 1	91.78 (91.78, 91.78)	4.92 (4.92, 4.92)	1.74 (1.74, 1.74)	1.51 (1.51, 1.51)	0.05 (0.05, 0.05)	0 (0, 0)	0 (0, 0)
Day 3	75.93 (75.77, 76.09)	5.52 (5.42, 5.62)	1.19 (1.14, 1.23)	0.92 (0.89, 0.95)	0.29 (0.27, 0.31)	0.23 (0.21, 0.25)	15.93 (15.80, 16.07)
Day 7	28.90 (28.74, 29.06)	2.09 (2.04, 2.15)	0.56 (0.53, 0.59)	0.49 (0.46, 0.51)	0.38 (0.36, 0.40)	1.19 (1.16, 1.23)	66.39 (66.22, 66.56)
Days since AKI Stage 1							
Day 1	0 (0, 0)	100 (100, 100)	0 (0, 0)	0 (0, 0)	0 (0, 0)	0 (0, 0)	0 (0, 0)
Day 3	59.82 (59.46, 60.18)	22.30 (21.92, 22.68)	4.34 (4.18, 4.50)	1.17 (1.10, 1.24)	0.63 (0.57, 0.69)	0.52 (0.48, 0.57)	11.23 (11.07, 11.40)
Day 7	30.23 (30.05, 30.41)	3.18 (3.09, 3.27)	1.19 (1.13, 1.25)	0.91 (0.86, 0.97)	0.80 (0.75, 0.86)	2.07 (2.00, 2.14)	61.62 (61.41, 61.82)
Days since AKI Stage 2							
Day 1	0 (0, 0)	0 (0, 0)	100 (100, 100)	0 (0, 0)	0 (0, 0)	0 (0, 0)	0 (0, 0)
Day 3	38.04 (37.44, 38.64)	26.43 (25.89, 26.98)	19.18 (18.57, 19.81)	6.06 (5.78, 6.34)	1.91 (1.75, 2.08)	1.31 (1.20, 1.43)	7.08 (6.87, 7.30)
Day 7	28.66 (28.43, 28.89)	5.08 (4.92, 5.24)	3.09 (2.93, 3.26)	3.18 (3.01, 3.35)	2.49 (2.34, 2.67)	4.67 (4.49, 4.87)	52.83 (52.50, 53.17)
Days Since AKI Stage 3 without KRT							
Day 1	0 (0, 0)	0 (0, 0)	0 (0, 0)	100 (100, 100)	0 (0, 0)	0 (0, 0)	0 (0, 0)
Day 3	17.58 (16.88, 18.31)	11.61 (11.06, 12.20)	15.50 (14.77, 16.26)	41.46 (40.28, 42.68)	7.82 (7.28, 8.41)	2.16 (1.88, 2.48)	3.86 (3.58, 4.17)
Day 7	19.51 (19.12, 19.90)	5.60 (5.36, 5.86)	5.69 (5.35, 6.06)	12.24 (11.60, 12.91)	9.69 (9.12, 10.30)	10.30 (9.77, 10.87)	36.97 (36.32, 37.63)
Days since AKI Stage 3 with KRT							
Day 1	0 (0, 0)	0 (0, 0)	0 (0, 0)	0 (0, 0)	100 (100, 100)	0 (0, 0)	0 (0, 0)
Day 3	0 (0, 0)	0 (0, 0)	0 (0, 0)	0 (0, 0)	92.83 (90.38, 95.34)	5.76 (3.87, 8.58)	1.41 (0.70, 2.81)
Day 7	0 (0, 0)	0 (0, 0)	0 (0, 0)	0 (0, 0)	68.71 (65.64, 71.93)	26.36 (23.51, 29.55)	4.93 (3.69, 6.58)

AKI, acute kidney injury; CI, confidence interval; KRT, kidney replacement therapy.

**Figure 2:**
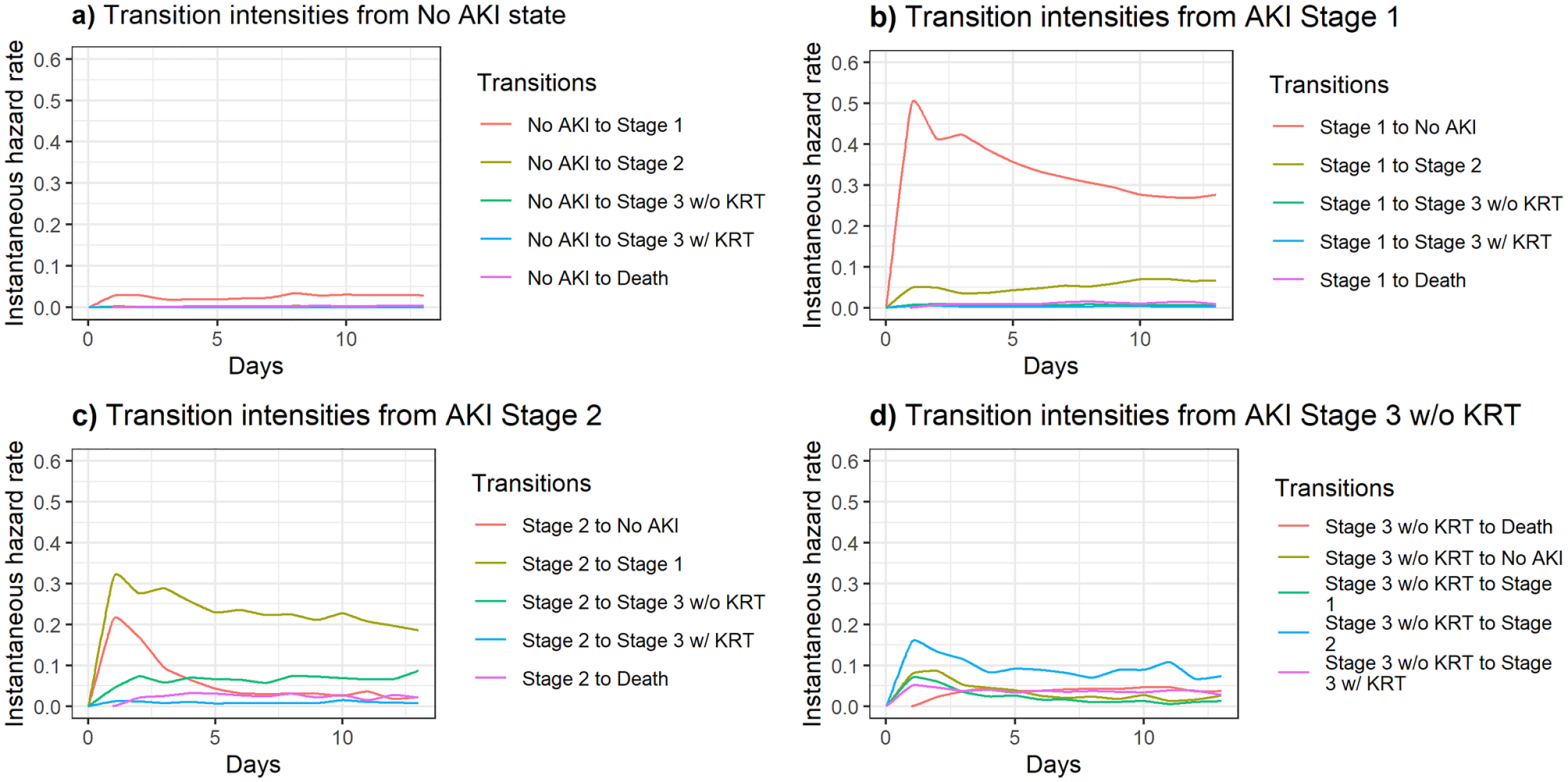
Instantaneous hazard rates of outcomes from clinical states in hospitalized patients based on multistate analysis.

Patients with No AKI diagnosis had the highest transition rates to AKI Stage 1 state throughout the hospitalization (Fig. 2a, Supplementary Fig. S3a). For those patients, first peak was observed within first two days, whereas the second peak transfer rate to AKI Stage 1 appeared near following completion of first week of the 14-day hospital stay. Though at much lower rates, transfer rates from No AKI to death repeated a similar pattern of peaks and lows as transfer rates to AKI Stage 1. AKI Stage 1 patients were more likely to start resolving in next day (Fig. 2b, Supplementary Fig. S3b). Among AKI Stage 1 patients, top transfer rates occurred for resolving state, while the second highest transfer rates were for advancing to Stage 2. These two top processes had slightly different timings for jumps occurring in a 14-day time period.

AKI Stage 2 cohort had the highest transition rates for regression to AKI Stage 1 (Fig. 2c, Supplementary Fig. S3c). Among patients with AKI Stage 2, hazard rates for transitioning towards resolution were observed higher than progressing to AKI Stage 3 without KRT until approximately day four and risk for progressing to AKI Stage 3 without KRT elevated and surpassed resolution hazard rates thereafter. AKI Stage 3 without KRT patients were more likely to transfer to AKI Stage 2 compared to transitions to AKI Stage 1 and No AKI states (Fig. 2d, Supplementary Fig. S3d). Transition rates from AKI Stage 3 without KRT to AKI Stage 2, AKI Stage 1, and No AKI were at their highest within the first two days. Instantaneous hazard rates for transitioning to death from AKI Stage 3 without KRT exceeded hazard rates for transitioning to resolution and AKI stages except AKI Stage 2 in the period following first week.

We fit a Cox model that involves transition-specific variables for each specified transfer from one given stage to another. We considered possible demographic and health condition indicators as risk factors. In that Cox model, the patient’s age, sex (female vs male), race (African American vs non–African American), CCI, and prolonged ICU stay were included. Age was dichotomized as age < 65 and age ≥ 65 years old, and CCI was dichotomized as CCI < 3 and CCI ≥ 3. Prolonged ICU was indicated with a length of stay longer than 48 h in ICU. We reported the Cox regression model coefficients, standard errors, and indication for significance of these variables in Supplementary Table S2. According to the results, these variables were statistically significant in a majority of the identified transitions.

**Figure 3:**
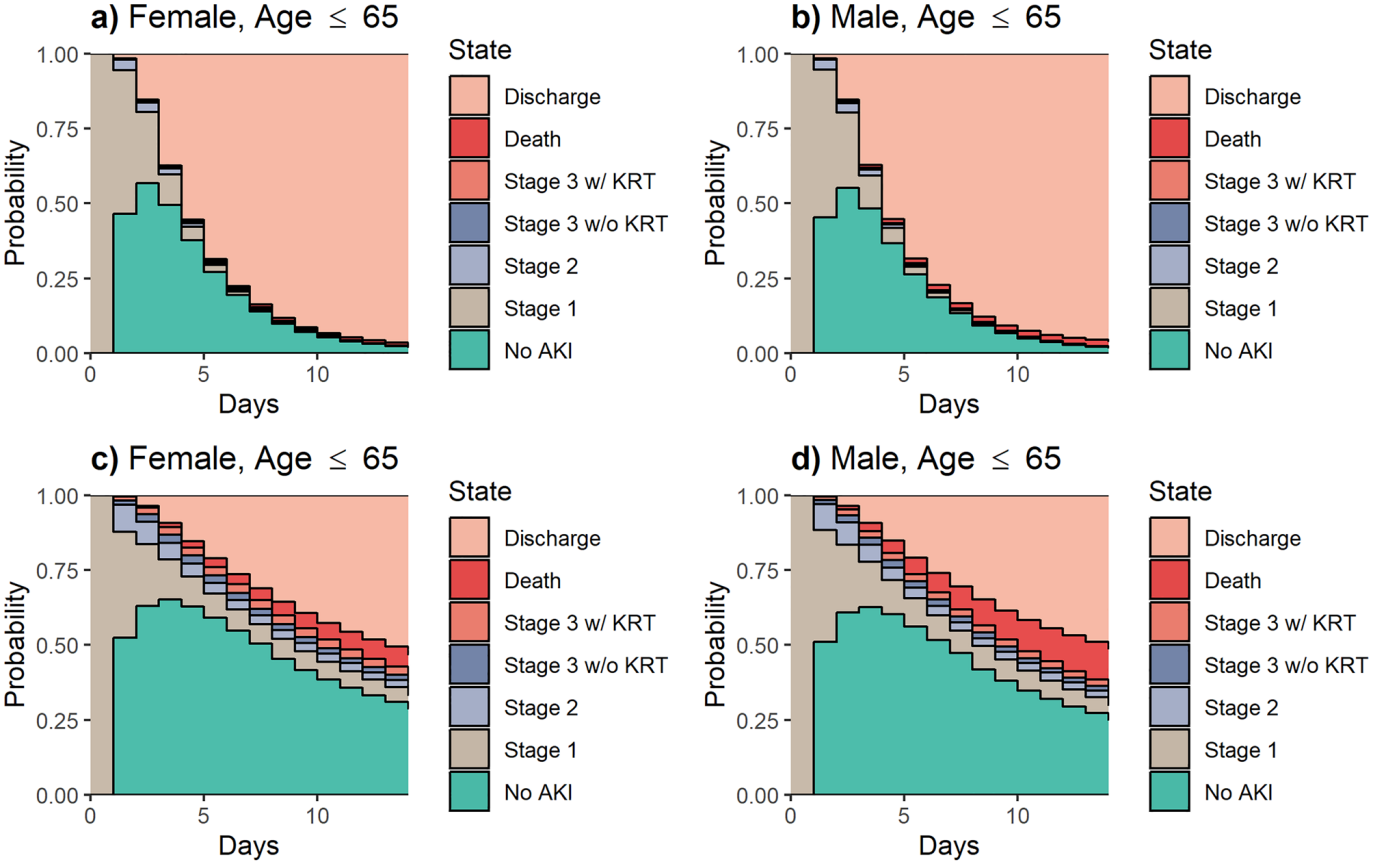
Proportion of non–African American patients estimated to be in each clinical state for Stage 1 AKI patients with CCI < 3 and ICU < 48 h (**a** and **b**) and Stage 1 AKI patients with CCI ≥ 3 and ICU stay ≥ 48 h (**c** and **d**).

Estimated percentages of patients for each state at any given day during hospital stay were mainly different with respect to their accompanied admission comorbidities and need of prolonged ICU stay (Fig. 3, Supplementary Figs. S4–S19). Among AKI Stage 1 cohort, patients accompanied with the most severe conditions (i.e., CCI ≥ 3 and ICU length of stay ≥ 48 h) had greater proportion of patients for sustained AKI Stage 1 severity and progression to higher stages of AKI and lower discharge percentage compared to patients with milder conditions (i.e., CCI < 3 and ICU length of stay < 48 h) (Fig. 3). Proportion of transfer to death condition from AKI states were more pronounced among male patients compared to female subjects throughout the 14-day period of hospital stay. Considering the cohorts with additional frail conditions, percentage of AKI Stage 3 without KRT progression was slightly higher for African American patients compared to non–African American subjects. In addition, similar to non-parametric analyses presented in this study, more advanced AKI stages were observed with higher tendency towards either maintaining current AKI condition or regressing to its neighbor AKI Stage when compared to AKI Stage 1 group (Supplementary Fig. S4–S19).

## Discussion

We considered a large cohort of hospitalized patients and retrospectively characterized subjects’ AKI trajectory by using multistate models that consider the cohort’s longitudinal outcomes. Specifically, we fit multistate models for a state-space framework that indicates the clinical status in addition to feasible transitions between them, and we described the transition dynamics in terms of hazard rates and transition probabilities^36^. In this cohort, any stage AKI was developed among 20% of the patients, where the majority of AKI outcomes were labeled as Stage 1.

In order to understand the transition processes between clinical states regarding the AKI status, death, and discharge, we modeled the patients’ experiences over the course of the first 14 days of hospitalization. Towards that aim, we first calculated Aalen-Johansen estimators via non-parametric multistate models and estimated the instantaneous hazard rates of each transition occurrence in addition to the probability of being in certain states at a given time point. This granular and time-dependent analyses demonstrated AKI Stage 1 as the AKI condition with the lowest tendency towards developing more advanced stages. To clarify, around 7 days following initiation of AKI Stage 1, 91.85% of this patient group had either resolved AKI or were discharged. In contrast, within 7 days following AKI Stage 2, the estimated proportion of patients either being discharged or with no AKI was 81.49%, and if regression to AKI Stage 1 was also included, the probability of an outcome with more desirable conditions increased to 86.57%.

We expanded the initial multistate analysis by modeling transitions’ hazard rates with Cox-type regression models to investigate the effect of a set of variables that are potentially associated with events of interest. According to Cox-type models, the variables indicate the severity of the overall condition of the subjects (i.e., CCI and prolonged ICU stay) heavily influenced the probability of being resolved or discharged. To clarify, near the end of a 14-day period of hospitalization with a Stage 1 AKI initiated at the beginning, patients admitted with a higher number of comorbidities and with prolonged ICU stay had a higher percentage for being at AKI Stage 1 state and transitioning to either progressed AKI stages or death. In line with that, No AKI patients admitted with a higher number of comorbidities and who stayed in the ICU longer than 48 h had a higher probability of being in either AKI stages or death.

The motivation for performing this study was that the clinical course of AKI for hospitalized patients had not been sufficiently described with multistate models that exploit granular and longitudinal structures, despite similar work having been performed for smaller patient populations. In a retrospective analysis of critically ill COVID-19 patients, a cohort of 367 subjects were considered and their AKI transitions were described with multistate models^37^. In that study, Lyons et al. presented the estimated probabilities of being in a specified clinical status where the AKI-related states rely on the worst AKI stage within 12-h blocks. Another recent multistate modeling application for investigating kidney disease progression was given for a cohort of 225 patients who were prescribed colistin^38^. In addition to AKI, a retrospective study for modeling transitions between CKD stages via multistate methods was given for a cohort of 117 hospitalized and non-hospitalized patients^39^. To our knowledge, our study is the first large-scale, granular application of multistate methods for describing the characteristics influencing the transitions towards progressed or regressed AKI stages in addition to discharge and death states. With the aid of a large, diverse cohort of hospitalized subjects, we utilized multistate methods in modeling the longitudinal outcomes regarding the patients’ AKI status.

The results from this study indicated that the hospital resource utilization and mortality were higher for more severe stages of AKI, showing 50%, 53%, and 88% needing to stay in ICU more than 48 h and 11%, 18%, and 45% with hospital death for Stage 2, Stage 3 without KRT, and Stage 3 AKI with KRT, respectively. Patients at more severe AKI stages were more likely to stay at that stage or progress to worse stages or die. This study provides us a better understanding of clinical course of AKI and characteristics influencing disease process and transition rates, and resource needs. It illustrates importance of precise and timely identification of patients at elevated risk for progression of AKI in order to provide the delivery of tailored treatments that can improve life quality and optimize resource planning.

Multistate models developed in this study output a probabilistic way to describe clinical course of AKI among hospitalized patients. Those estimations could be utilized in planning prevention decisions, resource usage, and timely intervention of AKI. Despite our use of a large and diverse cohort in building the multistate models, the cohort relies on a single institution. Consequently, this single-center design limits generalizability to other practice settings. We excluded encounters discharged within 24 h of admission, including those who died within 24 h due to not having sufficient transitions.

The timing precision of a transition relies on the serum creatinine measure date and time, however serum creatinine as a biomarker with a long half-life lags behind renal injury and recovery^40,41^. Future work could be based on defining AKI conditions considering more frequently obtained biomarkers with beyond suboptimal performance in diagnosing AKI, which allows more precise and timely monitoring as well as transition time records^42^.

## Conclusions

Harnessing the granular and longitudinal information processing capability of multistate models, we estimated possible pathways in clinical trajectories of AKI among hospitalized patients, thus stressing the ability of this approach to convey insights into AKI course from a probabilistic perspective. Moreover, the large and diverse cohort was expected to assist mitigating the bias in fitting the model. Precise and timely identification of patients at elevated risk for AKI progress or other terminal states may facilitate the delivery of tailored treatments that prevent adverse outcomes or foster kidney recovery to improve life quality and optimize resource planning.

### Supplementary Information

Supplementary Information.

## Data Availability

Data is available from the University of Florida Intelligent Clinical Care Center at ic3-center@ufl.edu and the University of Florida Integrated Data Repository at IRBDataRequest@ahc.ufl.edu for researchers who meet the criteria for access to confidential data and may require additional IRB approval (University of Florida IRB contact is Peter Iafrate, IRB Chair [iafrate@ufl.edu]). Author contact is Azra Bihorac (abihorac@ufl.edu).
