# Supplementary material for: Clinical courses of acute kidney injury in hospitalized patients: a multistate analysis
Source: Sci Rep. 2023 Oct 18;13:17781. doi: 10.1038/s41598-023-45006-5 (PMC10584933; doi:10.1038/s41598-023-45006-5)
Supplement: Supplementary file 1 — Supplementary Information. [file 41598_2023_45006_MOESM1_ESM.docx]

**Supplementary Online Content**

**Adiyeke E, Ren Y, Guan Z, Ruppert M, Rashidi P, Bihorac A, Ozrazgat-Baslanti T.**

**Clinical Courses of Acute Kidney Injury in Hospitalized Patients: A Multistate Analysis**

This supplementary material has been provided by the authors to give readers additional information about their work.

**Supplementary Material Table of Contents**

**Supplementary Figure S1.** Patient inclusion chart.

**Supplementary Figure S2.** State space diagram for clinical states used in multistate analysis (a) and daily transitions in the cohort for the first 14 days of admission (b).

**Supplementary Figure S3.** Instantaneous hazard rates**.**

**Supplementary Figure S4.** Proportion of non–African American patients estimated to be in each clinical state for AKI Stage 1 patients with CCI < 3 and ICU < 48 hours for 14 days.

**Supplementary Figure S5.** Proportion of non–African American patients estimated to be in each clinical state for AKI Stage 1 patients with CCI ≥ 3 and ICU stay ≥ 48 hours for 14 days.

**Supplementary Figure S6.** Proportion of non–African American patients estimated to be in each clinical state for AKI Stage 2 patients with CCI < 3 and ICU < 48 hours for 14 days.

**Supplementary Figure S7.** Proportion of non–African American patients estimated to be in each clinical state for AKI Stage 2 patients with CCI ≥ 3 and ICU stay ≥ 48 hours for 14 days.

**Supplementary Figure S8.** Proportion of non–African American patients estimated to be in each clinical state for AKI Stage 3 without KRT patients with CCI < 3 and ICU < 48 hours for 14 days.

**Supplementary Figure S9.** Proportion of non–African American patients estimated to be in each clinical state for AKI Stage 3 without KRT patients with CCI ≥ 3 and ICU stay ≥ 48 hours for 14 days.

**Supplementary Figure S10.** Proportion of non–African American patients estimated to be in each clinical state for No AKI patients with CCI < 3 and ICU < 48 hours for 14 days.

**Supplementary Figure S11.** Proportion of non–African American patients estimated to be in each clinical state for No AKI patients with CCI ≥ 3 and ICU stay ≥ 48 hours for 14 days.

**Supplementary Figure S12.** Proportion of African American patients estimated to be in each clinical state for AKI Stage 1 patients with CCI < 3 and ICU < 48 hours for 14 days.

**Supplementary Figure S13.** Proportion of African American patients estimated to be in each clinical state for AKI Stage 1 patients with CCI ≥ 3 and ICU stay ≥ 48 hours for 14 days.

**Supplementary Figure S14.** Proportion of African American patients estimated to be in each clinical state for AKI Stage 2 patients with CCI < 3 and ICU < 48 hours for 14 days.

**Supplementary Figure S15.** Proportion of African American patients estimated to be in each clinical state for AKI Stage 2 patients with CCI ≥ 3 and ICU stay ≥ 48 hours for 14 days.

**Supplementary Figure S16.** Proportion of African American patients estimated to be in each clinical state for AKI Stage 3 without KRT patients with CCI < 3 and ICU < 48 hours for 14 days.

**Supplementary Figure S17.** Proportion of African American patients estimated to be in each clinical state for AKI Stage 3 without KRT patients with CCI ≥ 3 and ICU stay ≥ 48 hours for 14 days.

**Supplementary Figure S18.** Proportion of African American patients estimated to be in each clinical state for No AKI patients with CCI < 3 and ICU < 48 hours for 14 days.

**Supplementary Figure S19.** Proportion of African American patients estimated to be in each clinical state for No AKI patients with CCI ≥ 3 and ICU stay ≥ 48 hours for 14 days.

**Supplementary Table S1.** Detailed cohort characteristics and outcomes stratified by worst AKI severity.

**Supplementary Table S2.** Regression coefficients for the model adjusted for age, sex, race, CCI ≥ 3, and ICU ≥ 48 hours (standard errors are given in parenthesis).


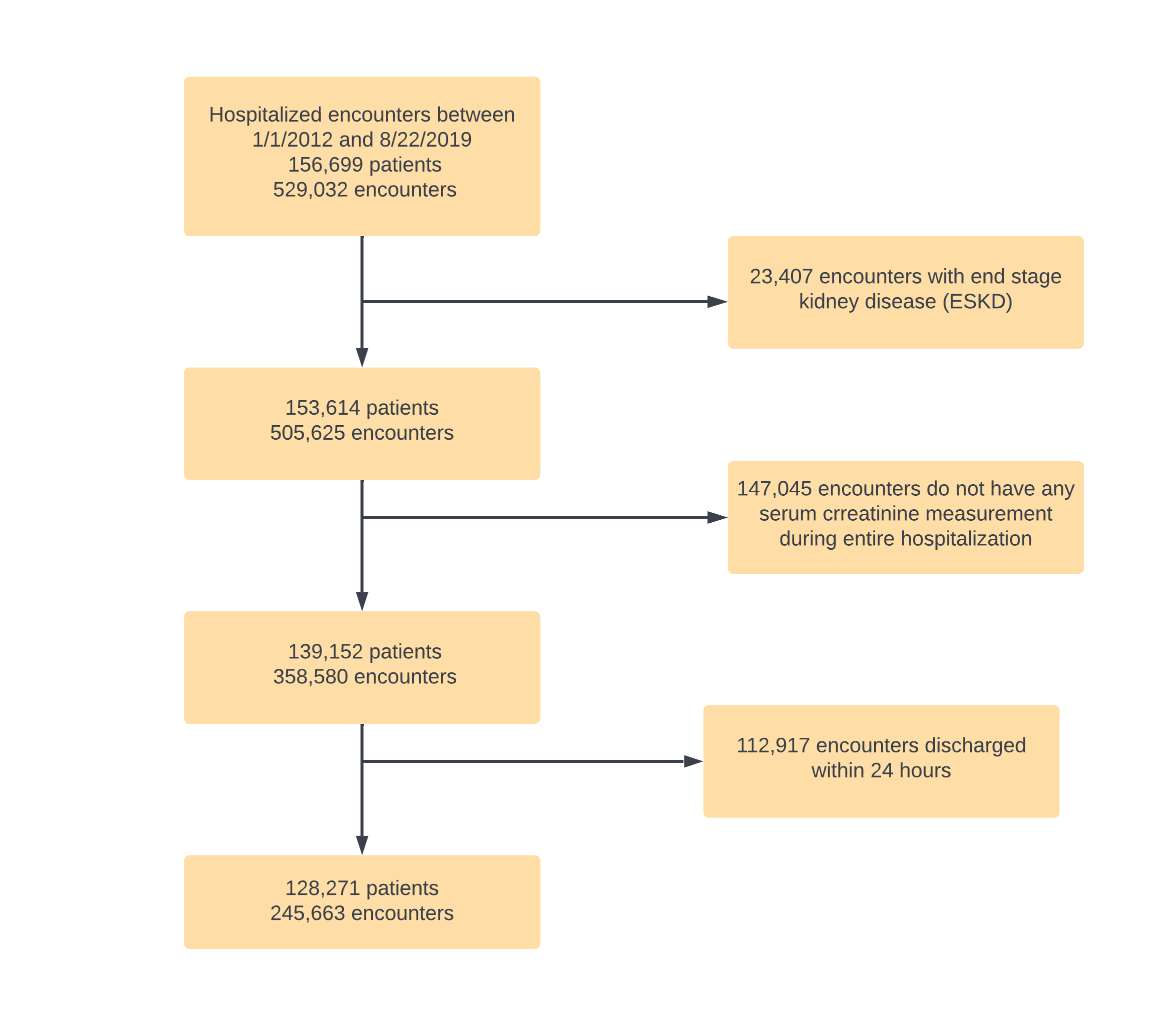


Supplementary Figure S1: Patient inclusion chart


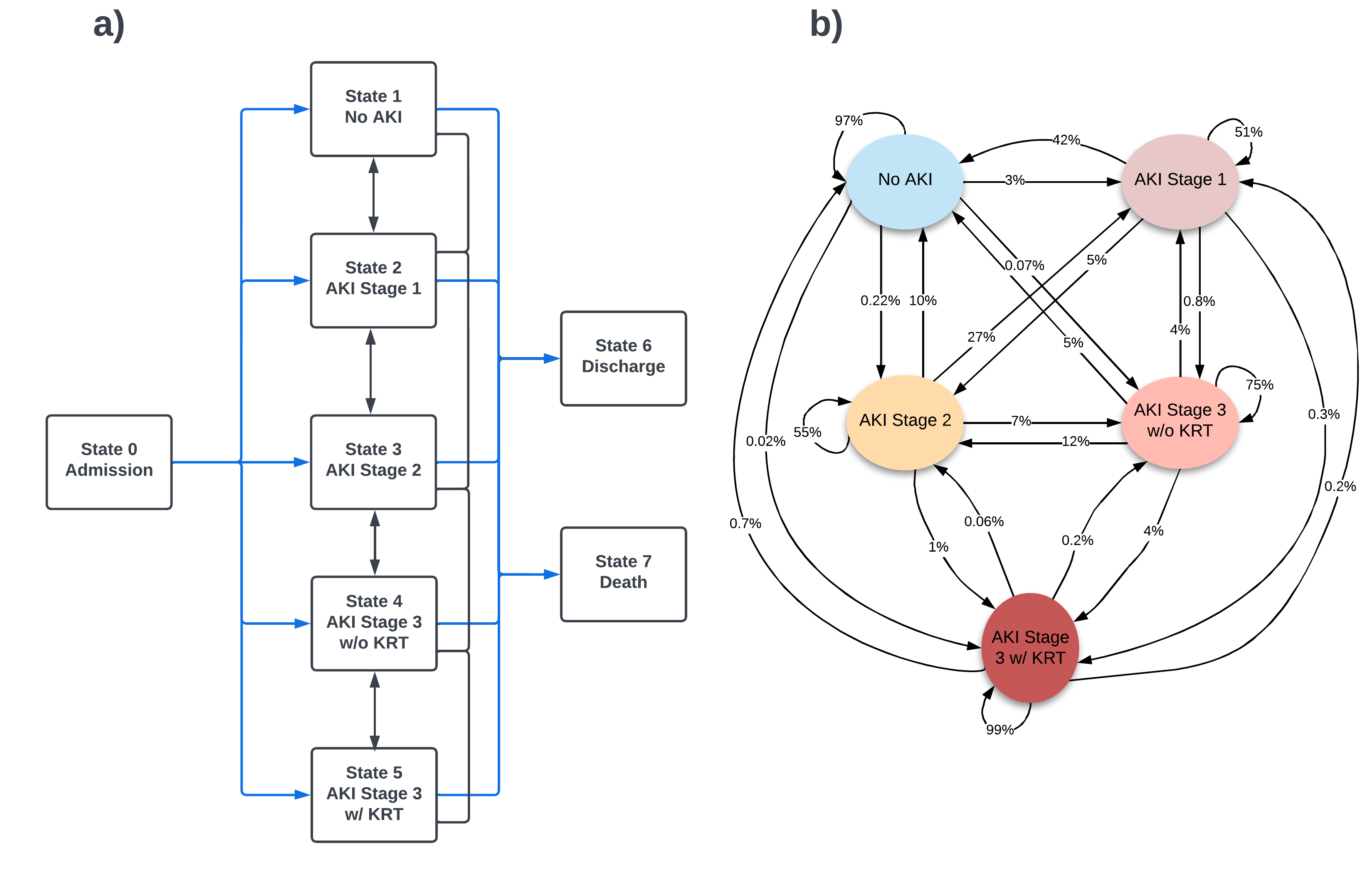


Supplementary Figure S2: State space diagram for clinical states used in multistate analysis (a) and daily transitions in the cohort for the first 14 days of admission (b).


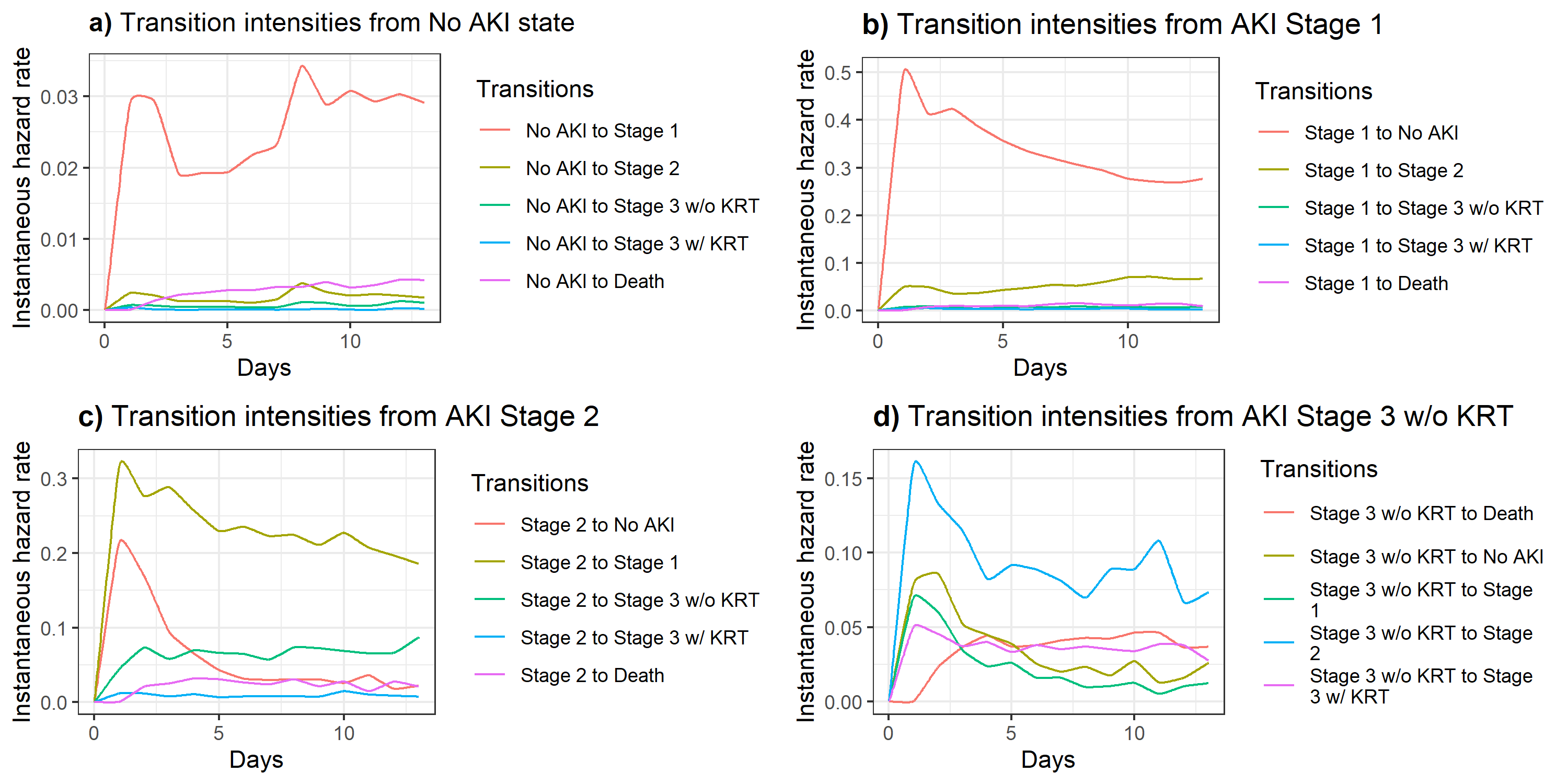


Supplementary Figure S3: Instantaneous hazard rates**.**


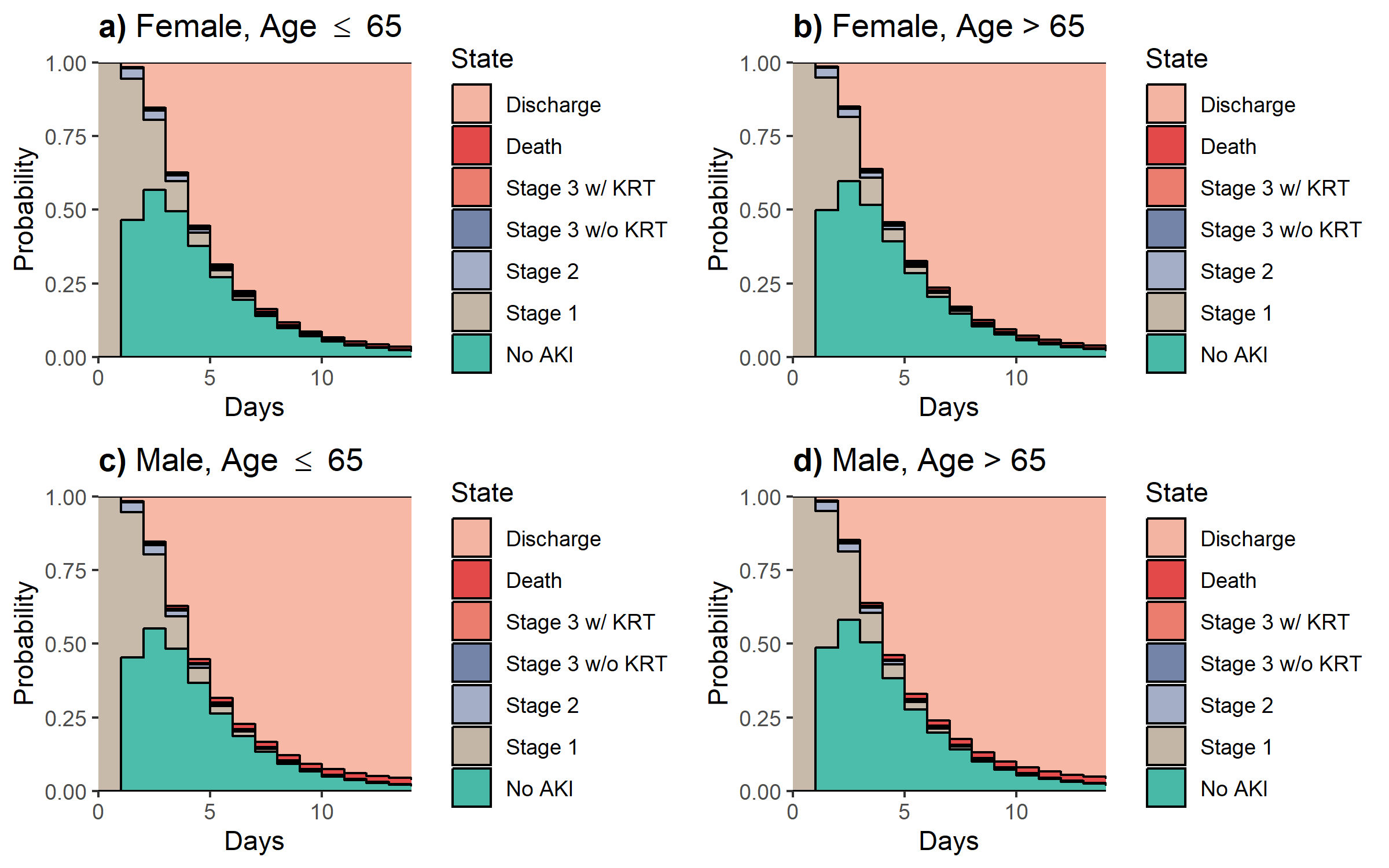


Supplementary Figure S4: Proportion of non–African American patients estimated to be in each clinical state for AKI Stage 1 patients with CCI < 3 and ICU < 48 hours for 14 days.


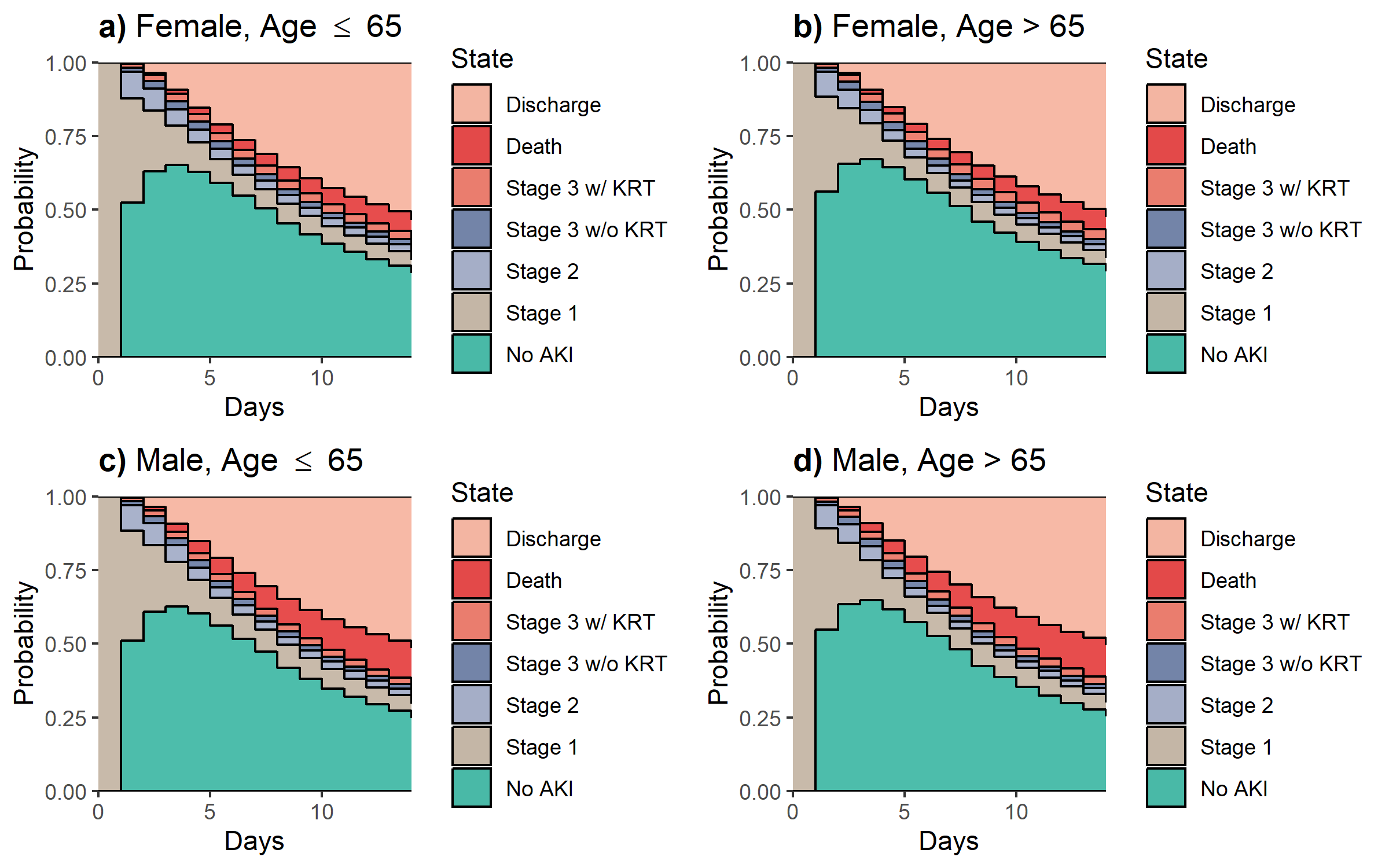


Supplementary Figure S5: Proportion of non–African American patients estimated to be in each clinical state for AKI Stage 1 patients with CCI ≥ 3 and ICU stay ≥ 48 hours for 14 days.


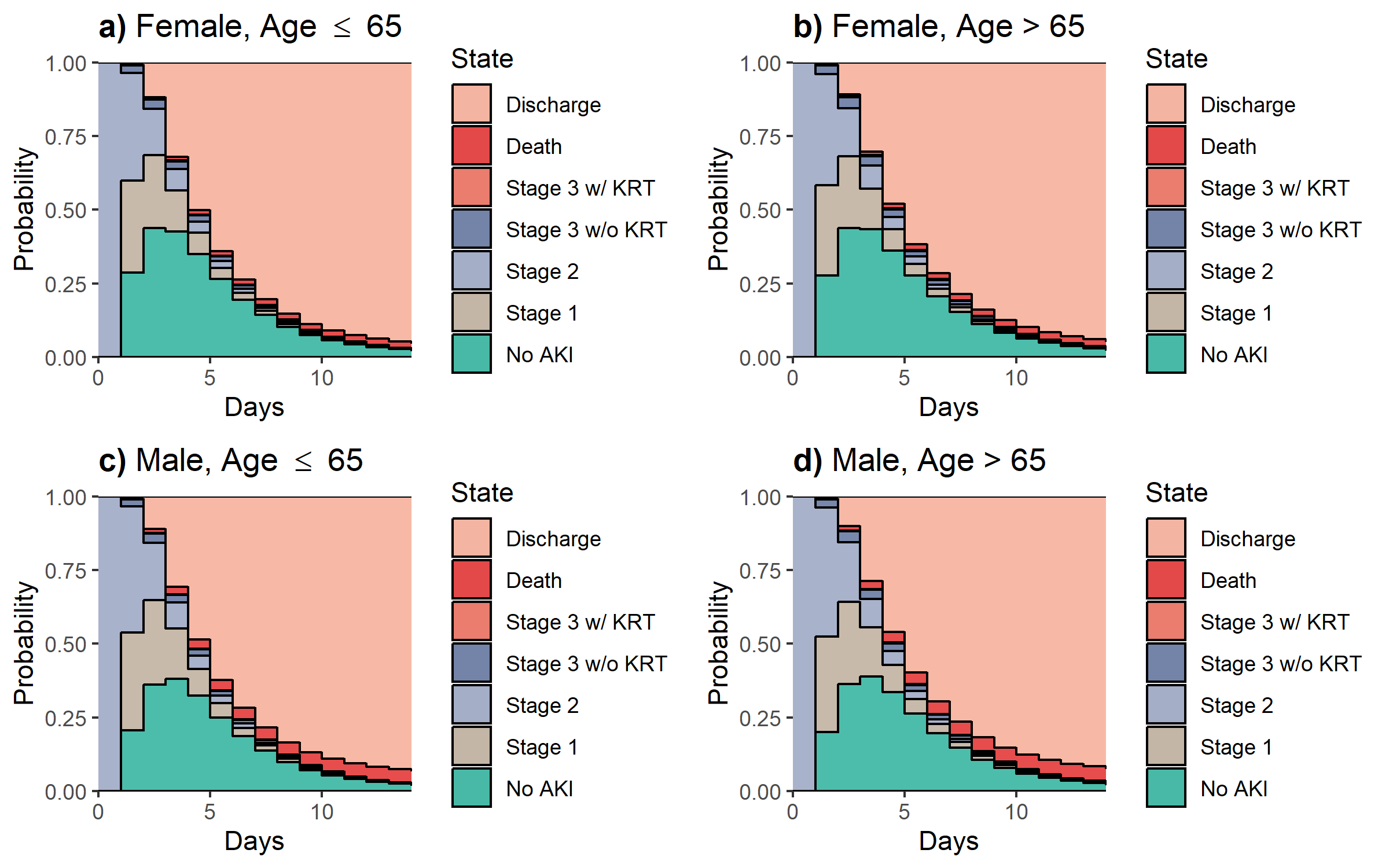


Supplementary Figure S6: Proportion of non–African American patients estimated to be in each clinical state for AKI Stage 2 patients with CCI < 3 and ICU < 48 hours for 14 days.


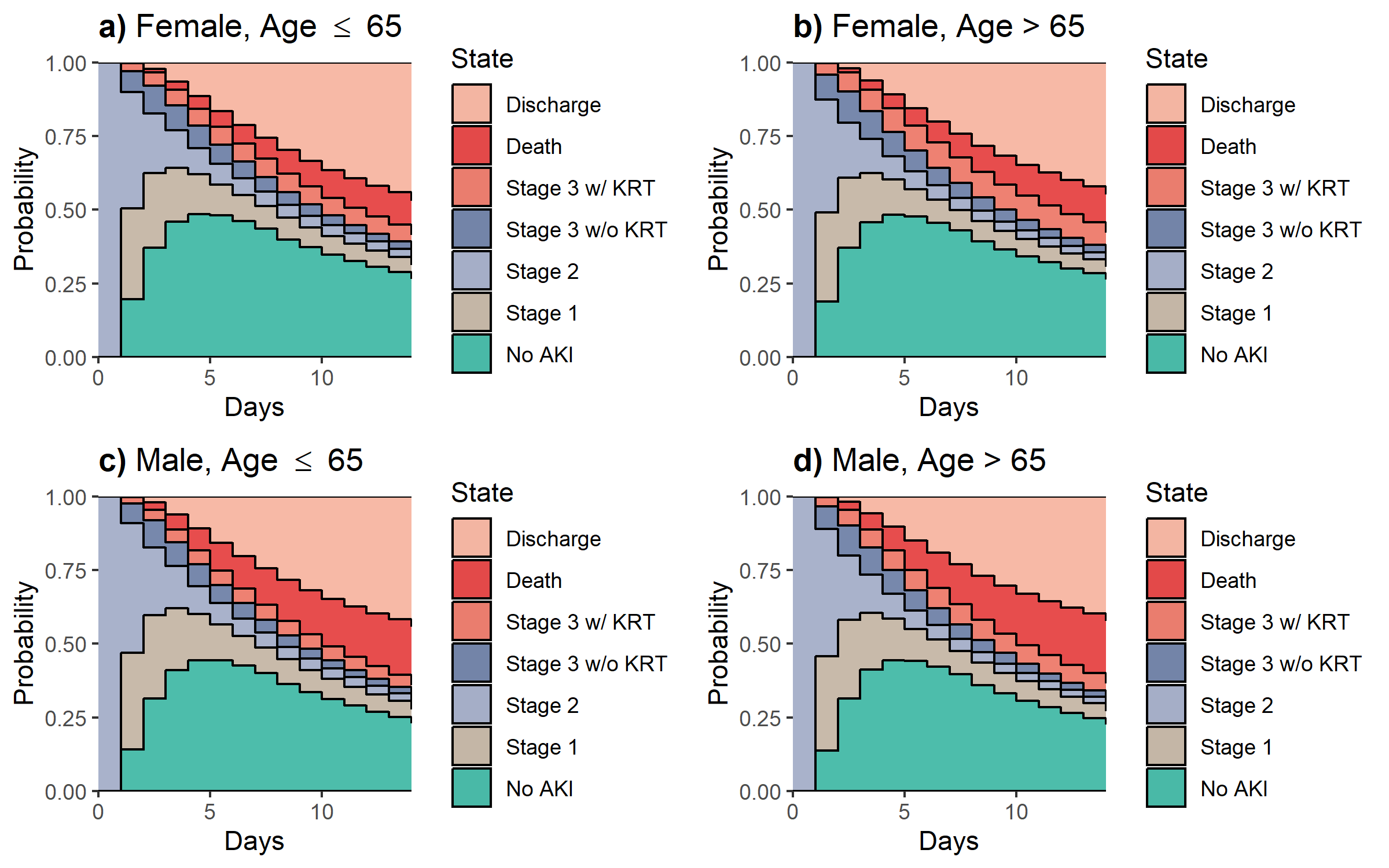


Supplementary Figure S7: Proportion of non–African American patients estimated to be in each clinical state for AKI Stage 2 patients with CCI ≥ 3 and ICU stay ≥ 48 hours for 14 days.


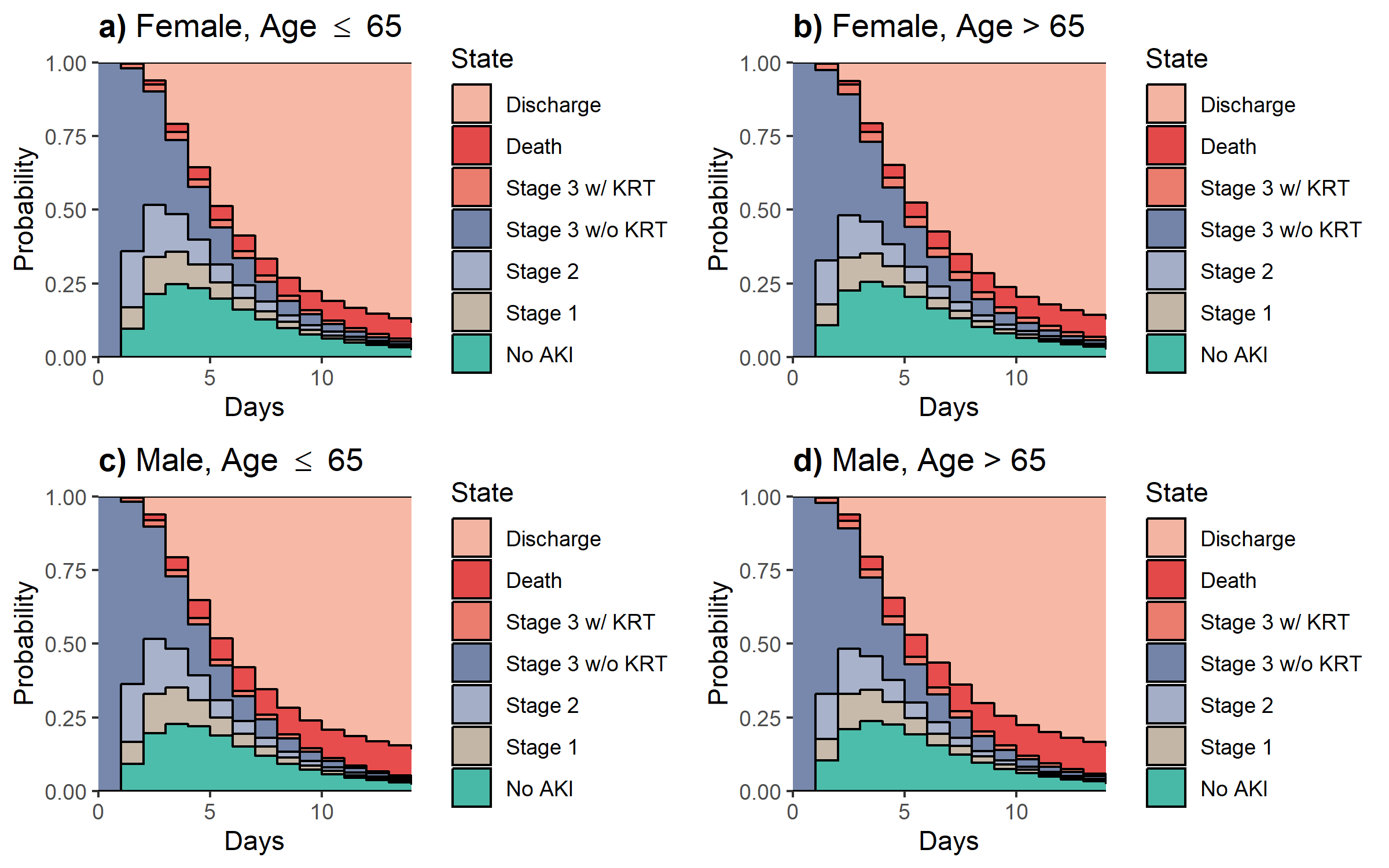


Supplementary Figure S8: Proportion of non–African American patients estimated to be in each clinical state for AKI Stage 3 without KRT patients with CCI < 3 and ICU < 48 hours for 14 days.

*
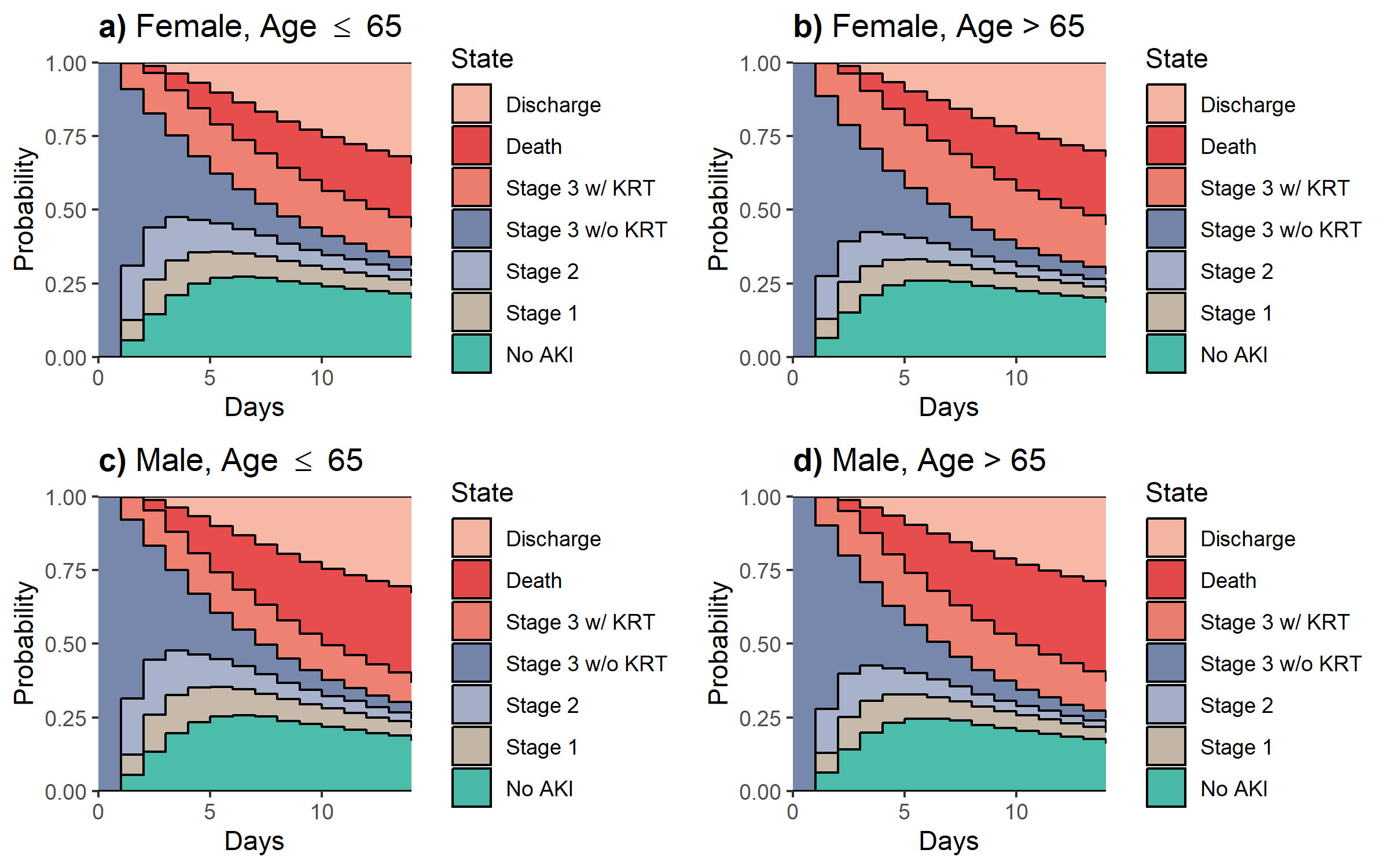
*

Supplementary Figure S9: Proportion of non–African American patients estimated to be in each clinical state for AKI Stage 3 without KRT patients with CCI ≥ 3 and ICU stay ≥ 48 hours for 14 days.


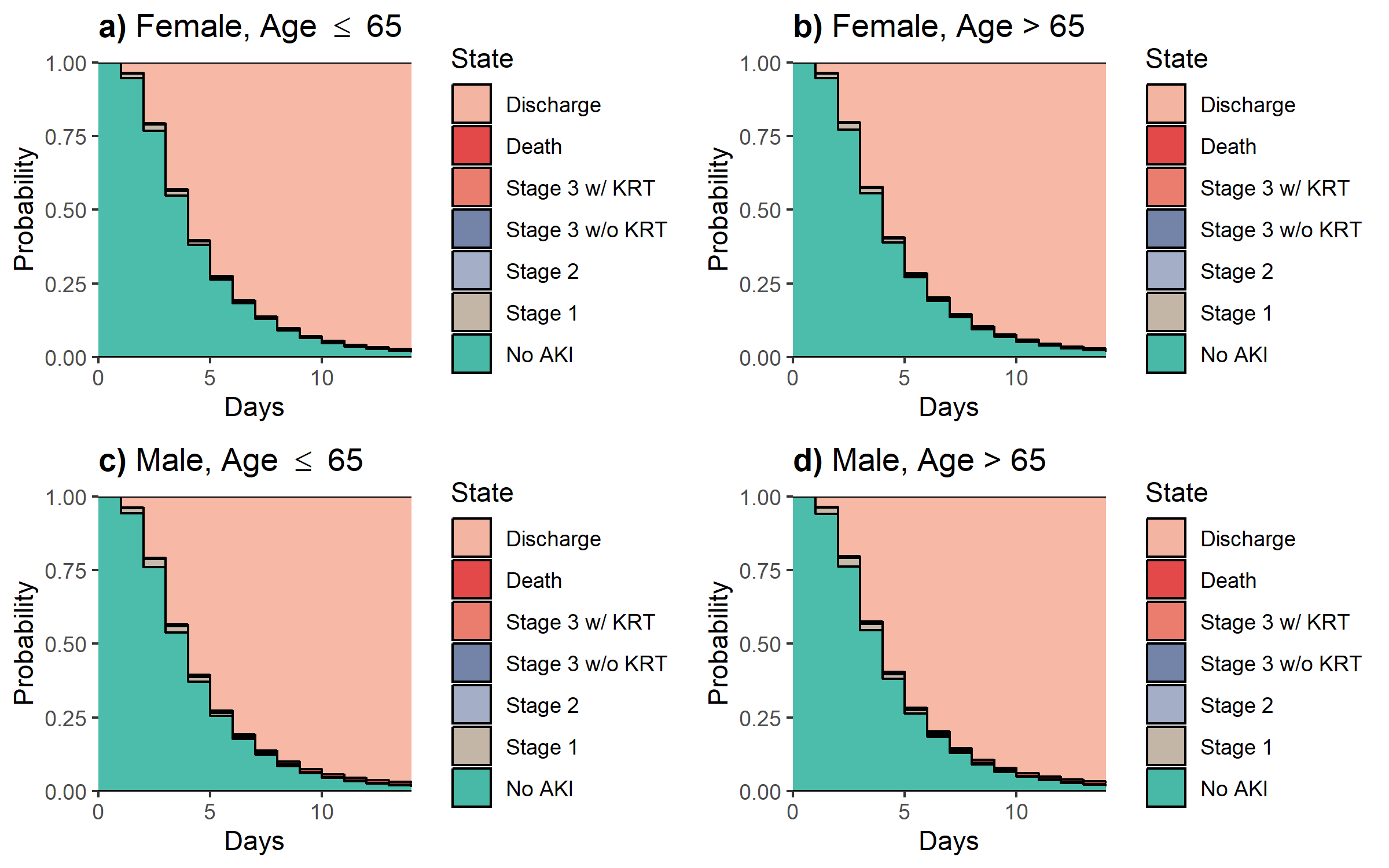


Supplementary Figure S10: Proportion of non–African American patients estimated to be in each clinical state for No AKI patients with CCI < 3 and ICU < 48 hours for 14 days.


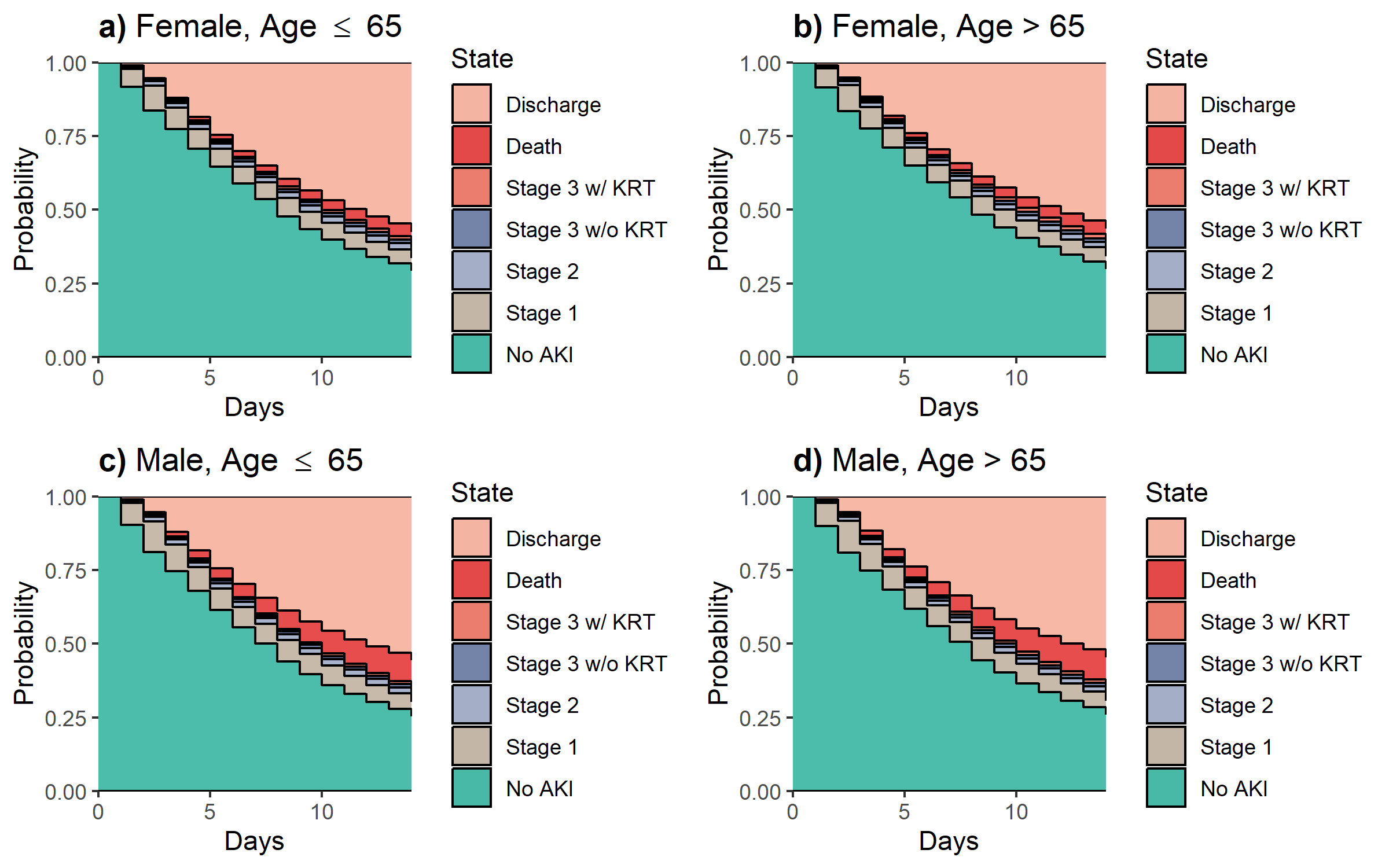


Supplementary Figure S11: Proportion of non–African American patients estimated to be in each clinical state for No AKI patients with CCI ≥ 3 and ICU stay ≥ 48 hours for 14 days.

**
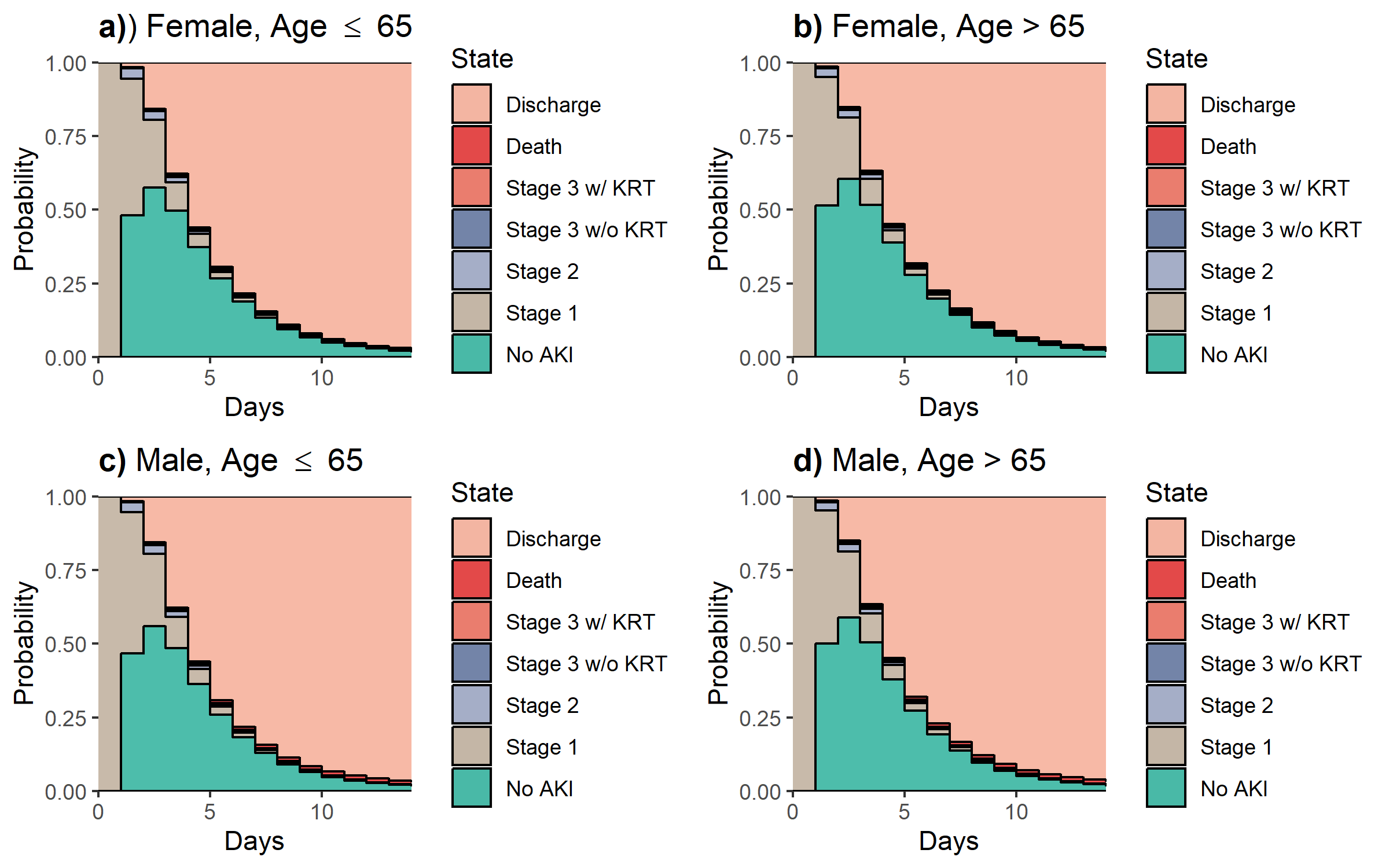
**

Supplementary Figure S12: Proportion of African American patients estimated to be in each clinical state for AKI Stage 1 patients with CCI < 3 and ICU < 48 hours for 14 days.


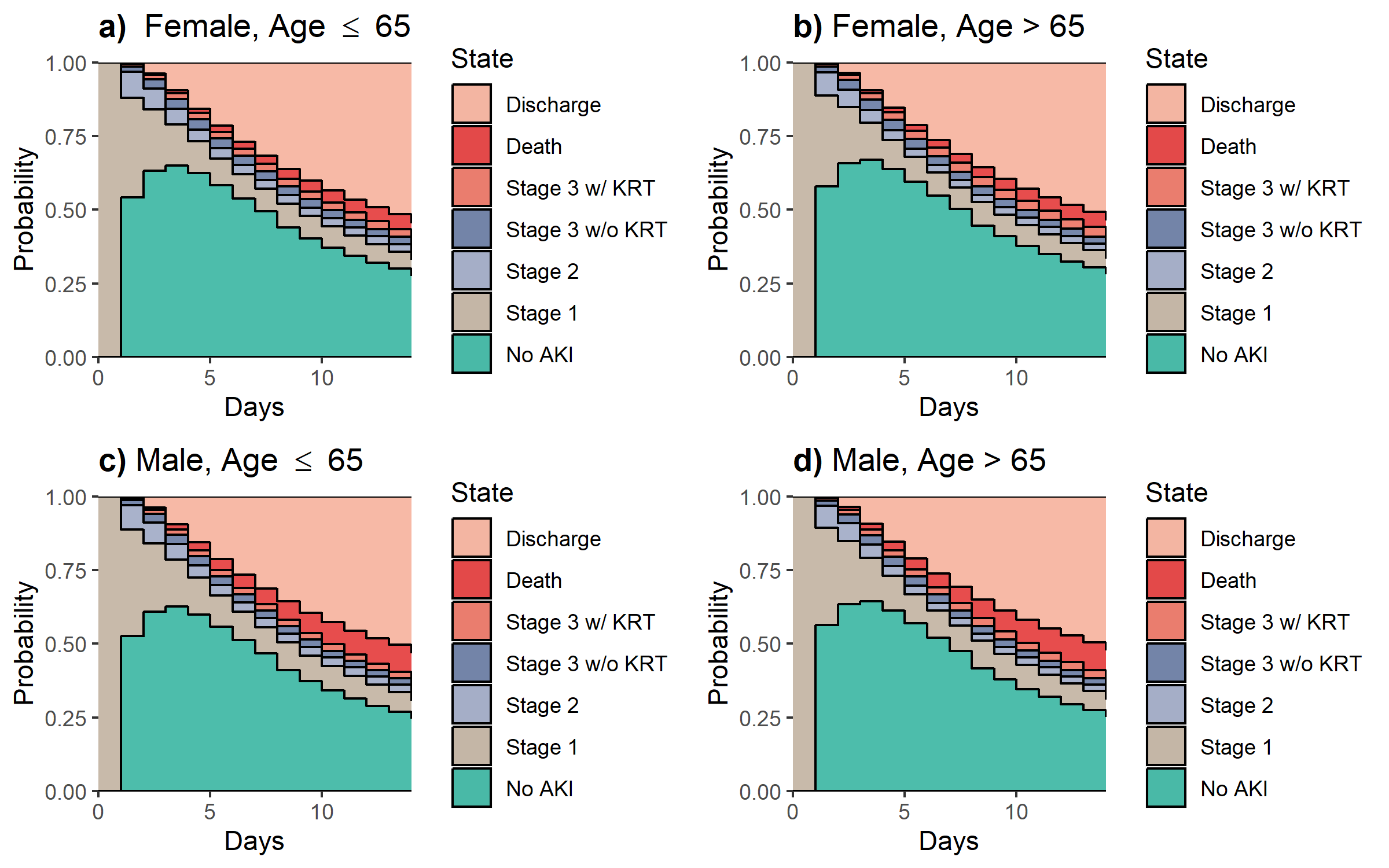


Supplementary Figure S13: Proportion of African American patients estimated to be in each clinical state for AKI Stage 1 patients with CCI ≥ 3 and ICU stay ≥ 48 hours for 14 days.


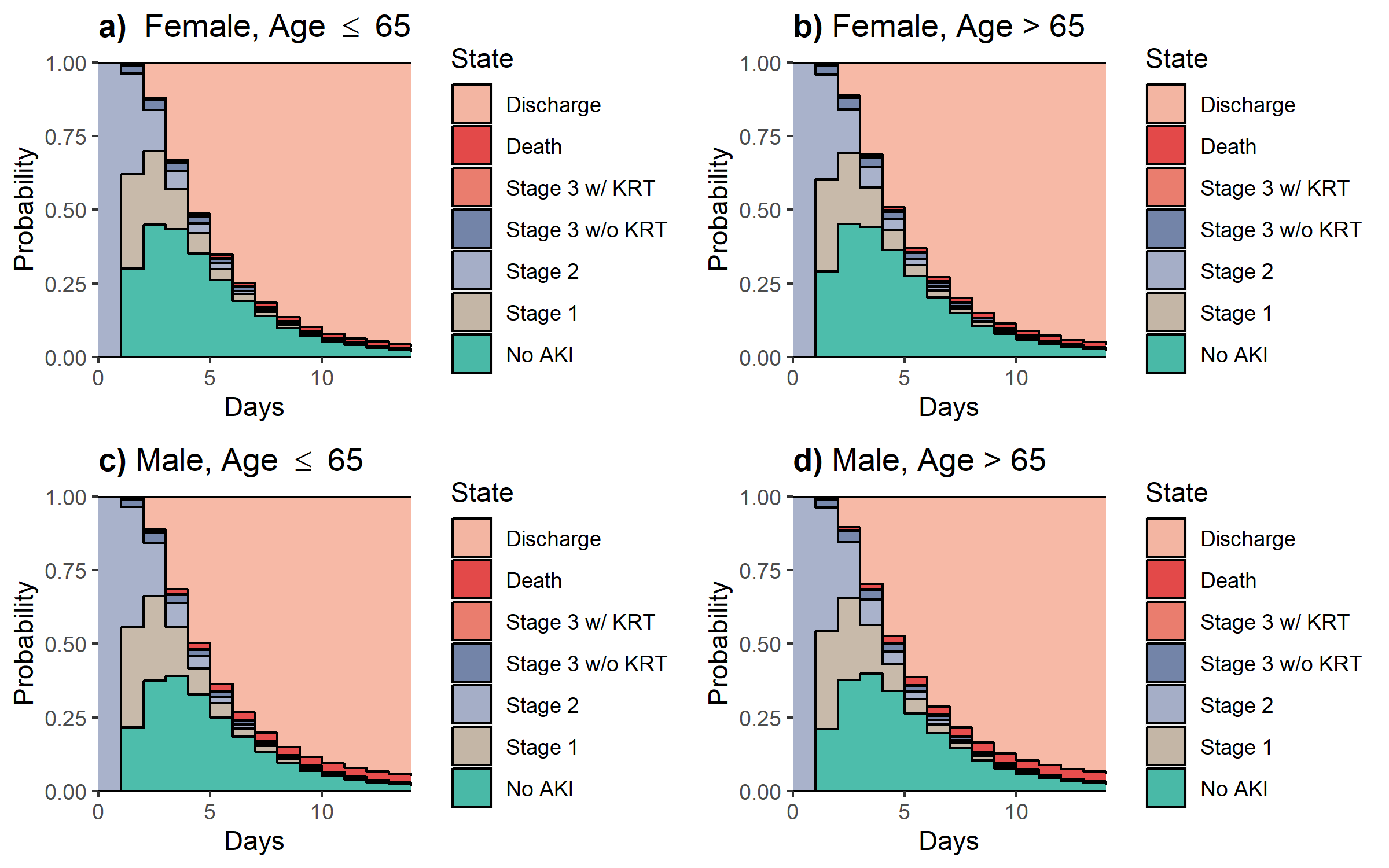


Supplementary Figure S14: Proportion of African American patients estimated to be in each clinical state for AKI Stage 2 patients with CCI < 3 and ICU < 48 hours for 14 days.


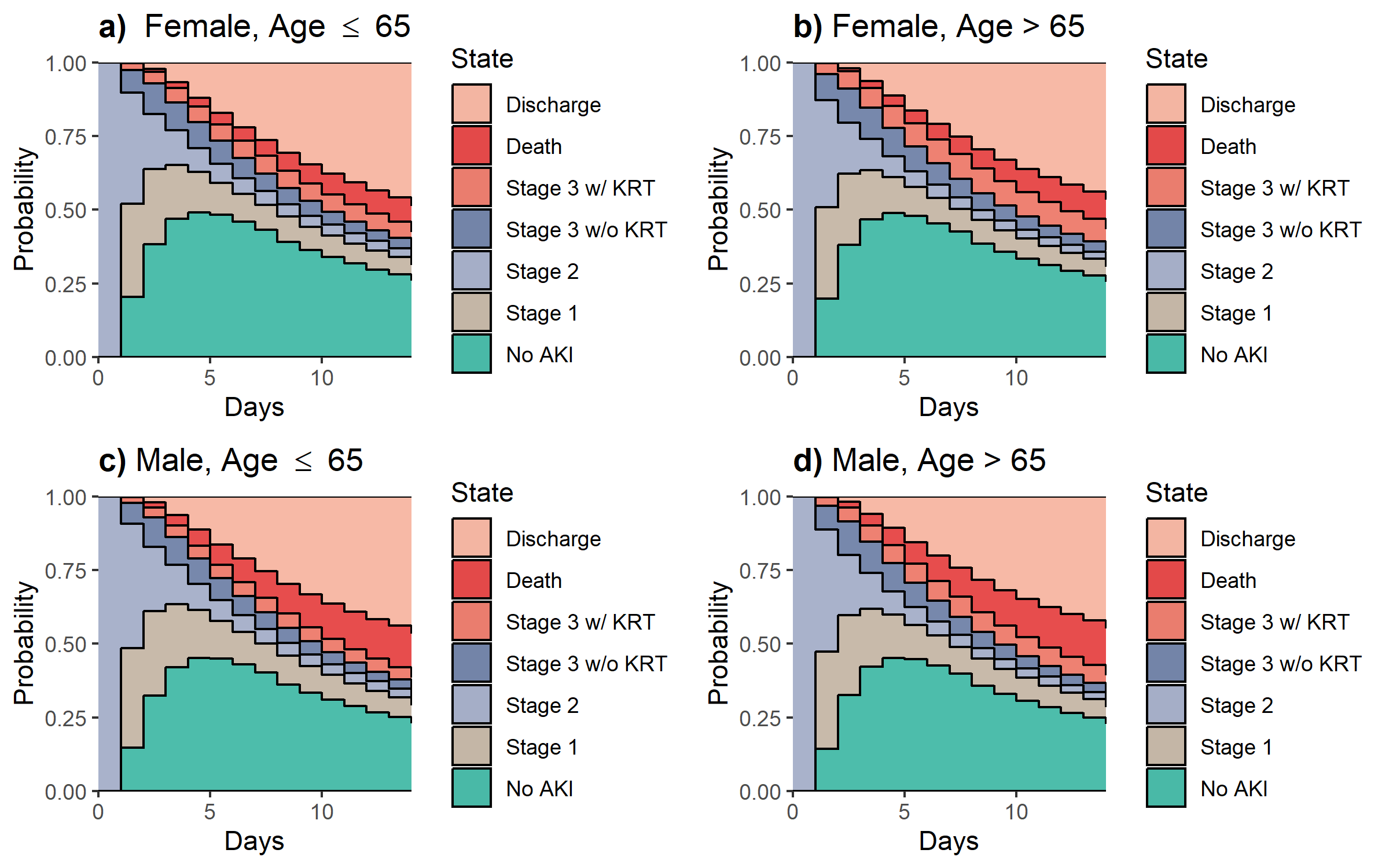


Supplementary Figure S15: Proportion of African American patients estimated to be in each clinical state for AKI Stage 2 patients with CCI ≥ 3 and ICU stay ≥ 48 hours for 14 days.


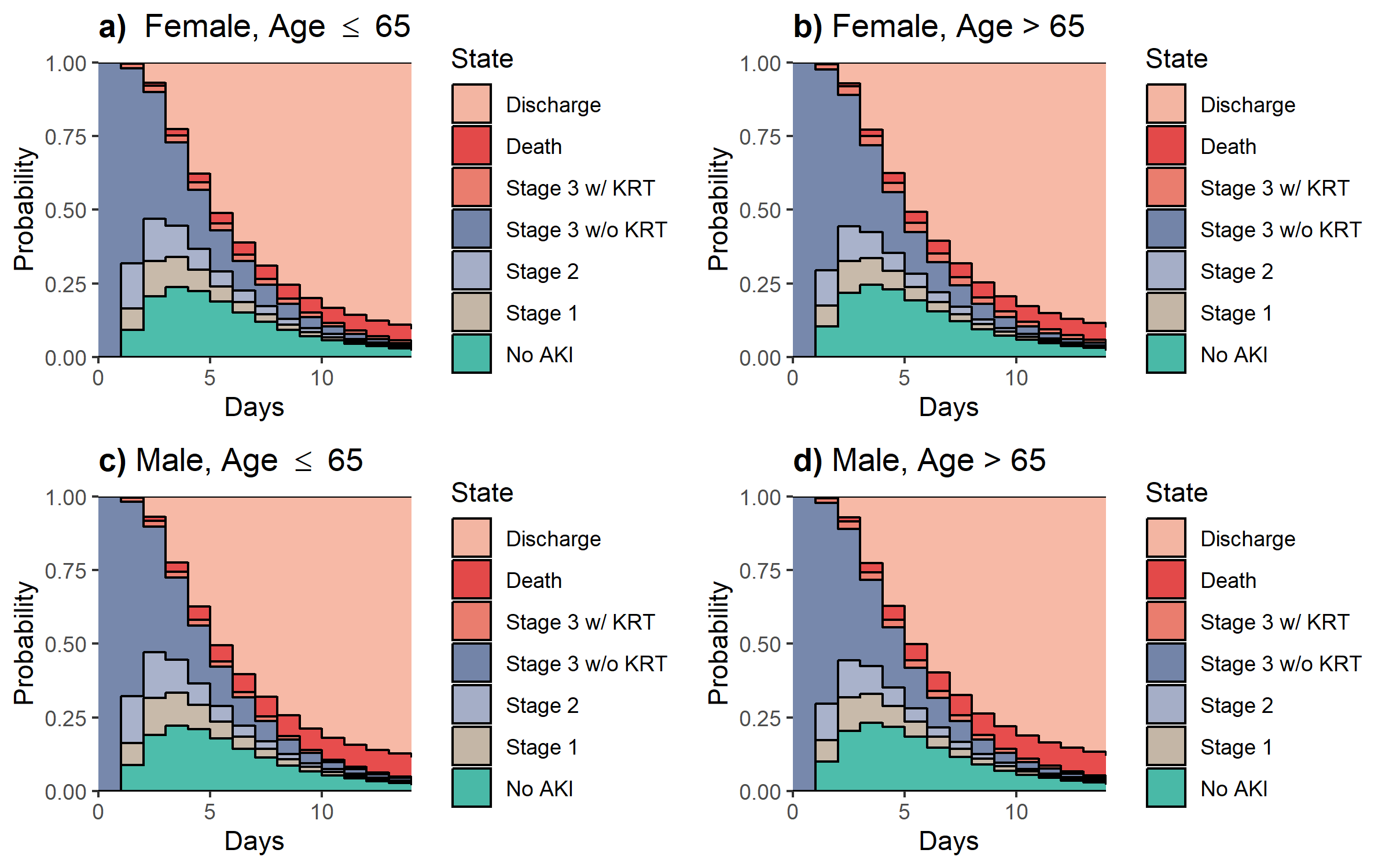


Supplementary Figure S16: Proportion of African American patients estimated to be in each clinical state for AKI Stage 3 without KRT patients with CCI < 3 and ICU < 48 hours for 14 days.


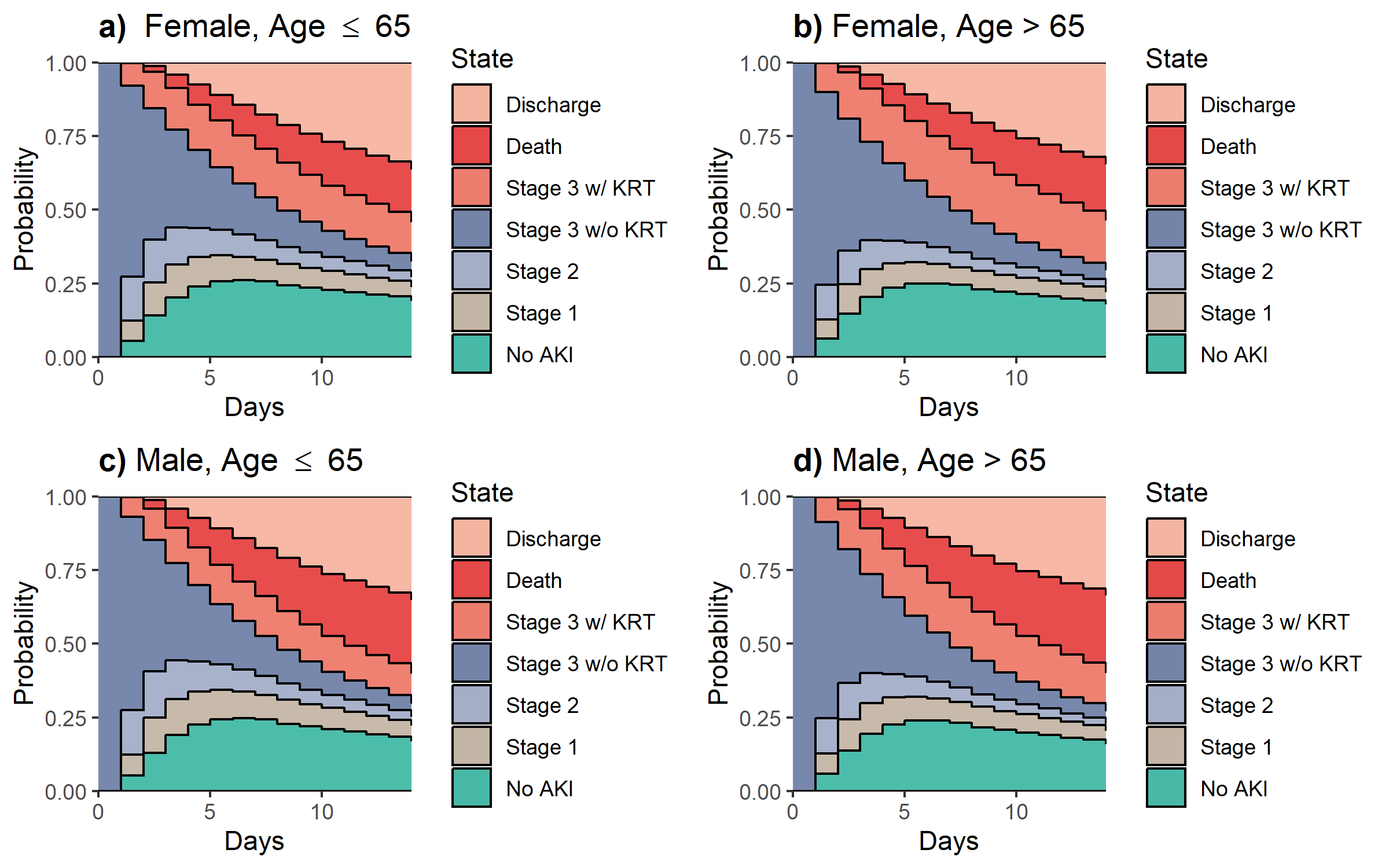


Supplementary Figure S17: Proportion of African American patients estimated to be in each clinical state for AKI Stage 3 without KRT patients with CCI ≥ 3 and ICU stay ≥ 48 hours for 14 days.


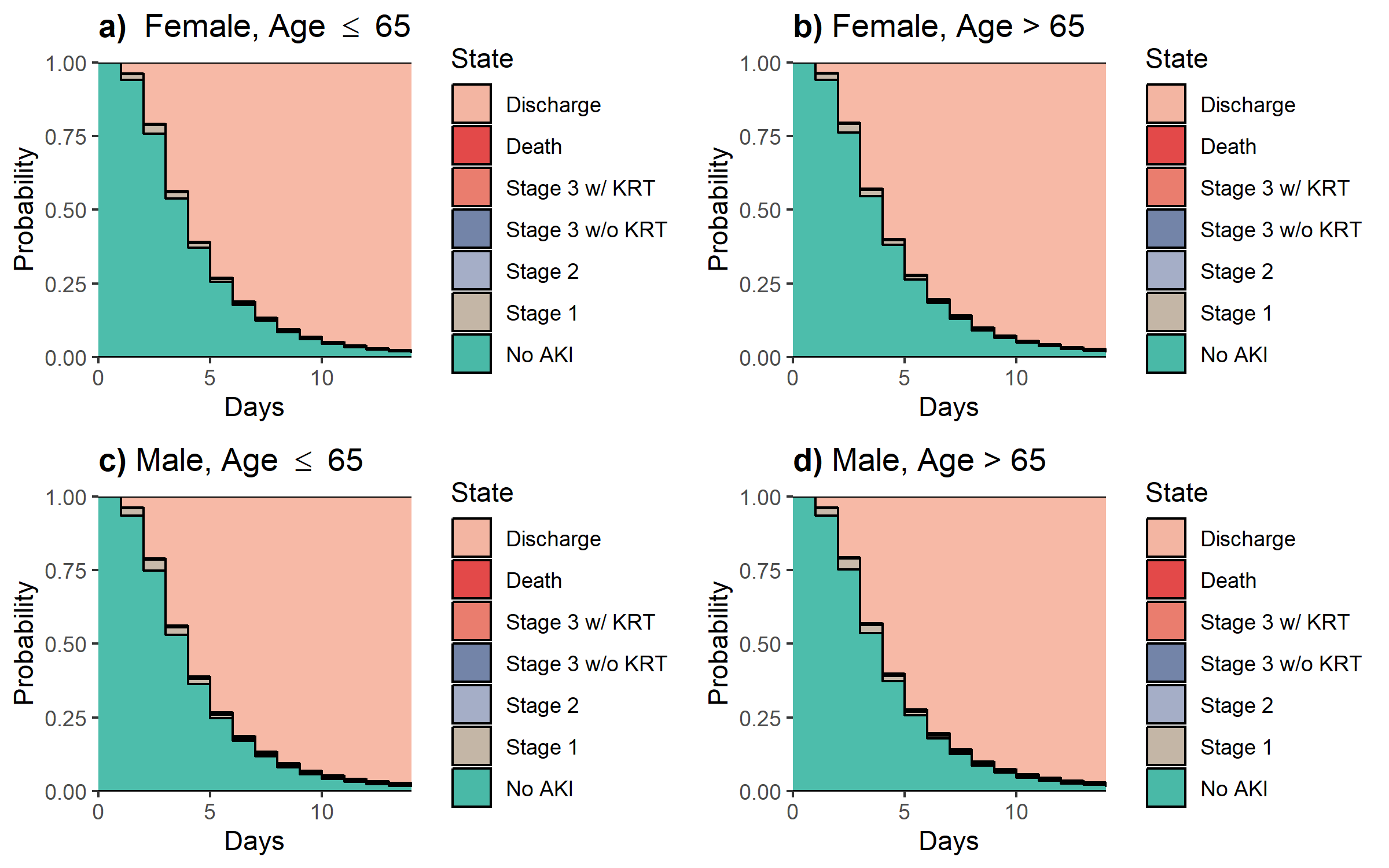


Supplementary Figure S18: Proportion of African American patients estimated to be in each clinical state for No AKI patients with CCI < 3 and ICU < 48 hours for 14 days.


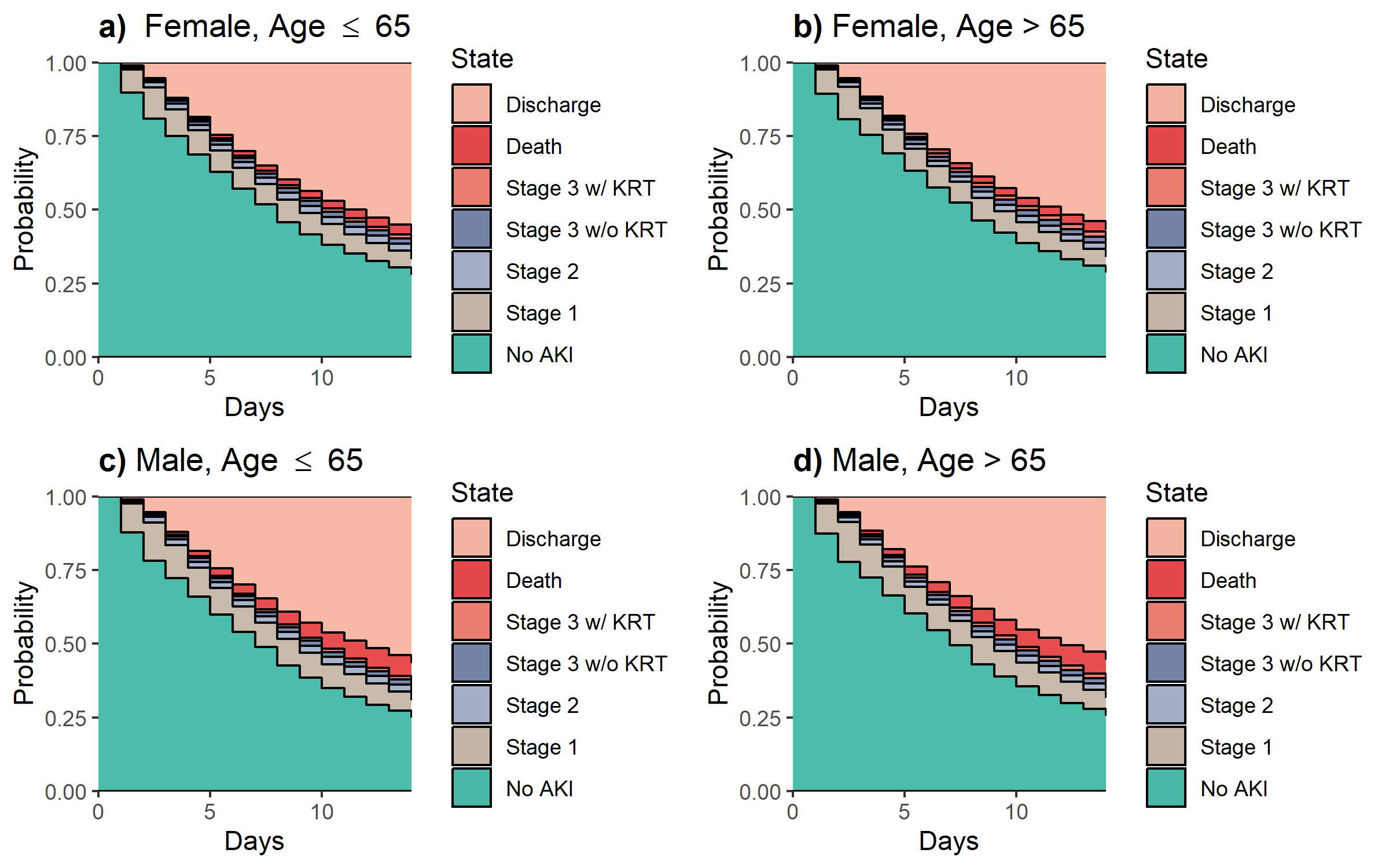


Supplementary Figure S19: Proportion of African American patients estimated to be in each clinical state for No AKI patients with CCI ≥ 3 and ICU stay ≥ 48 hours for 14 days.

**Supplementary Table S1:** Detailed cohort characteristics and outcomes stratified by worst AKI severity.

| **Features** | **No AKI** | **Stage 1** | **Stage 2** | **Stage 3 w/o KRT** | **Stage 3 w/ KRT** |
| --- | --- | --- | --- | --- | --- |
| Number of encounters, n | 195,581 | 33,260 | 8,779 | 6,004 | 2,039 |
| Age in years, mean (SD) | 55 (18) | **61 (17)** | **61 (17)** | **60 (16)** | **59 (15)** |
| Female, n (%) | 103,009 (53) | **16,451 (49)** | 4,580 (52) | **2,855 (48)** | **802 (39)** |
| Ethnicity, n (%) |  |  |  |  |  |
| Hispanic | 8,257 (4) | **1,216 (4)** | **283 (3)** | 209 (3) | 73 (4) |
| Non-Hispanic | 185,275 (95) | **31,712 (95)** | **8,401 (96)** | 5,725 (95) | 1,903 (93) |
| Missing | 2,035 (1) | 329 (1) | 95 (1) | 69 (1) | **63 (3)** |
| Race, n (%) |  |  |  |  |  |
| African American | 38,959 (20) | **7,434 (22)** | **1,882 (21)** | **1,543 (26)** | 423 (21) |
| Other | 11,970 (6) | **1,731 (5)** | **446 (5)** | **297 (5)** | 110 (5) |
| Missing | 1,156 (1) | **257 (1)** | **78 (1)** | **55 (1)** | **58 (3)** |
| White | 143,496 (73) | **23,838 (72)** | 6,373 (73) | **4,109 (68)** | 1,448 (71) |
| Smoking Status, n (%) |  |  |  |  |  |
| Never | 79,081 (40) | **12,287 (37)** | **3,205 (37)** | **2,207 (37)** | **670 (33)** |
| Former | 57,846 (30) | **11,632 (35)** | **2,928 (33)** | **1,925 (32)** | **700 (34)** |
| Current | 41,291 (21) | **5,966 (18)** | **1,615 (18)** | **1,145 (19)** | **309 (15)** |
| Missing | 17,363 (9) | **3,375 (10)** | **1,031 (12)** | **727 (12)** | **360 (18)** |
| CKD, n (%) |  |  |  |  |  |
| CKD | 40,573 (21) | **12,988 (39)** | **2,909 (33)** | **3,002 (50)** | **11,94 (59)** |
| Comorbid conditions on admission date, n (%) |  |  |  |  |  |
| Charlson comorbidity index ≥ 3, n (%) | 51,877 (27) | **15,140 (46)** | **4,153 (47)** | **3,208 (53)** | **1,273 (62)** |
| Charlson comorbidity index, median (IQR) | 1 (0, 3) | **2 (1, 4)** | **2 (1, 4)** | **3 (1, 5)** | **3 (2, 5)** |
| Myocardial Infarction | 25,208 (13) | **5,587 (17)** | **1,305 (15)** | **879 (15)** | 255 (13) |
| Congestive Heart Failure | 46,314 (24) | **10,336 (31)** | **2,654 (30)** | **1,780 (30)** | **597 (29)** |
| Peripheral Vascular  Disease | 38,929 (20) | **8,429 (25)** | **2,099 (24)** | **1,440 (24)** | **464 (23)** |
| Diabetes | 55,732 (28) | **11,556 (35)** | **2,910 (33)** | **1,918 (32)** | 626 (31) |
| Hypertension | 122,746 (63) | **22,561 (68)** | **5,964 (68)** | **4,012 (67)** | 1,281 (63) |
| Cancer | 50,099 (26) | **8,939 (27**) | 2,336 (27) | 1,593 (27) | **452 (22)** |
| Metastatic Carcinoma | 16,469 (8) | 2,929 (9) | 788 (9) | 547 (9) | **126 (6)** |
| Moderate-Severe Liver Disease | 41,734 (21) | **7,696 (23)** | **2,207 (25)** | **1,475 (25)** | 454 (22) |
| Reference creatinine, mean (SD) | 0.84 (0.32) | **0.97 (0.47)** | **0.85 (0.30)** | **1.53 (1.61)** | **1.63 (1.58)** |
| Mechanical ventilation days, median (IQR) | 2 (2, 4) | **3 (2, 7)** | **4 (2, 9)** | **5 (3, 10)** | **9 (4, 18)** |
| Length of stay (days), median (IQR) | 3 (2, 6) | **7 (4, 12)** | **8 (4, 16)** | **8 (5, 16)** | **21 (10, 35)** |
| ICU length of stay (days), median (IQR) | 3 (2, 6) | **5 (3, 10)** | **7 (4, 13)** | **6 (4, 13)** | **15 (7, 28)** |
| ICU length of stay ≥ 48 hours, n (%) | 29,987 (15) | **12,625 (38)** | **4,385 (50)** | **3,133 (52)** | **1,803 (88)** |
| Hospital mortality, n (%) | 2,361 (1) | **1,593 (5)** | **993 (11)** | **1,097 (18)** | **920 (45)** |
| Abbreviations: AKI, acute kidney injury; CKD, chronic kidney disease; ICU, intensive care unit; IQR, interquartile range; KRT, kidney replacement therapy; SD, standard deviation.  Bold values in eTable 1 indicate a Bonferroni corrected p-value ≤ 0.05 compared to No AKI group. | | | | | |

**Supplementary Table S2.** Regression coefficients for the model adjusted for age, sex, race, CCI ≥ 3, and ICU ≥ 48 hours (standard errors are given in parenthesis)

| **Transition** | **Age** | **Sex** | **Race** | **CCI≥3** | **ICU≥48 hours** | **Transition** | **Age** | **Sex** | **Race** | **CCI≥3** | **ICU≥48 hours** |
| --- | --- | --- | --- | --- | --- | --- | --- | --- | --- | --- | --- |
| **Admission to No AKI** | **-0.02 (<0.001)** | **0.01 (<0.001)** | **-0.01 (<0.001)** | **-0.05 (<0.001)** | **-0.07 (0.00)** | **Stage 2 to Stage 1** | **0.06 (0.02)** | -0.02 (0.02) | 0.03 (0.03) | -0.04 (0.02) | 0.03 (0.02) |
| **Admission to AKI Stage 1** | **0.31 (0.02)** | **-0.11 (0.02)** | **0.10 (0.02)** | **0.37 (0.02)** | **0.48 (0.02)** | **Stage 2 to Stage 3 w/o KRT** | -0.08 (0.05) | **0.15 (0.05)** | 0.06 (0.06) | **0.33 (0.05)** | **0.72 (0.06)** |
| **Admission to AKI Stage 2** | **0.19 (0.03)** | **-0.12 (0.03)** | **0.11 (0.04)** | **0.52 (0.03)** | **0.79 (0.03)** | **Stage 2 to Stage 3 w/ KRT** | -0.24 (0.13) | **0.40 (0.14)** | -0.08 (0.17) | **0.20 (0.13)** | **2.71 (0.28)** |
| **Admission to AKI Stage 3 w/o KRT** | -0.02 (0.03) | **0.12 (0.03)** | **0.38 (0.04)** | **0.73 (0.03)** | **1.01 (0.03)** | **Stage 2 to Death** | **0.68 (0.09)** | 0.00 (0.09) | **-0.34 (0.12)** | **0.43 (0.09)** | -0.11 (0.09) |
| **Admission to AKI Stage 3 w/ KRT** | **-0.66 (0.20)** | 0.35 (0.18) | -0.17 (0.23) | 0.95 (0.18) | **2.40 (0.21)** | **Stage 2 to Discharge** | 0.04 (0.05) | **-0.12 (0.05)** | 0.03 (0.06) | **-0.45 (0.05)** | **-1.51 (0.06)** |
| **No AKI to Stage 1** | **0.21 (0.01)** | **0.05 (0.01)** | **0.25 (0.01)** | **0.63 (0.01)** | **0.73 (0.01)** | **Stage 3 w/o KRT to No AKI** | -0.04 (0.07) | 0.12 (0.07) | -0.04 (0.07) | **-0.19 (0.06)** | **-0.33 (0.07)** |
| **No AKI to Stage 2** | -0.03 (0.04) | **-0.09 (0.04)** | **0.12 (0.05)** | **0.38 (0.04)** | **1.32 (0.05)** | **Stage 3 w/o KRT to Stage 1** | 0.02 (0.08) | -0.04 (0.08) | 0.01 (0.09) | -0.05 (0.08) | -0.02 (0.08) |
| **No AKI to Stage 3 w/o KRT** | -0.07 (0.08) | **0.15 (0.07)** | **0.50 (0.08)** | **0.54 (0.07)** | **1.08 (0.08)** | **Stage 3 w/o KRT to Stage 2** | 0.03 (0.04) | **-0.24 (0.04)** | **-0.22 (0.05)** | **-0.15 (0.04)** | **0.11 (0.04)** |
| **No AKI to Stage 3 w/ KRT** | 0.21 (0.16) | 0.15 (0.16) | 0.25 (0.19) | **0.86 (0.16)** | **2.96 (0.25)** | **Stage 3 w/o KRT to Stage 3 w/ KRT** | -0.14 (0.07) | **0.24 (0.07)** | -0.13 (0.08) | 0.11 (0.07) | **1.55 (0.10)** |
| **No AKI to Death** | **0.96 (0.04)** | 0.00 (0.04) | **-0.39 (0.06)** | **0.52 (0.04)** | **0.76 (0.04)** | **Stage 3 w/o KRT to Death** | **0.36 (0.08)** | 0.02 (0.08) | **-0.25 (0.10)** | **0.47 (0.09)** | **0.20 (0.08)** |
| **No AKI to Discharge** | **0.01 (<0.001)** | **-0.02 (0.00)** | **0.02 (0.01)** | **-0.26 (<0.001)** | **-1.08 (0.01)** | **Stage 3 w/o KRT to Discharge** | -0.02 (0.06) | 0.11 (0.06) | **0.28 (0.06)** | **-0.15 (0.06)** | **-1.50 (0.07)** |
| **Stage 1 to No AKI** | **-0.03 (0.01)** | **0.07 (0.01)** | **0.03 (0.01)** | 0.01 (0.01) | **0.11 (0.01)** | **Stage 3 w/ KRT to No AKI** | -0.12 (0.28) | 0.02 (0.27) | -0.08 (0.33) | -0.11 (0.27) | 0.48 (0.61) |
| **Stage 1 to Stage 2** | -0.05 (0.03) | **-0.10 (0.03)** | -0.05 (0.04) | **0.23 (0.03)** | **0.72 (0.03)** | **Stage 3 w/ KRT to Stage 1** | -0.04 (0.48) | -0.30 (0.45) | 0.51 (0.48) | 0.47 (0.48) | -0.05 (0.76) |
| **Stage 1 to Stage 3** | -0.09 (0.08) | 0.04 (0.08) | **0.33 (0.09)** | **0.56 (0.08)** | **0.88 (0.09)** | **Stage 3 w/ KRT to Stage 2** | 0.40 (0.97) | -1.64 (1.12) | **-6.41 (0.55)** | 0.73 (1.14) | **6.22 (0.55)** |
| **Stage 1 to Stage 3 w/ KRT** | -0.08 (0.12) | 0.08 (0.13) | **-0.34 (0.16)** | **0.60 (0.13)** | **2.52 (0.21)** | **Stage 3 w/ KRT to Stage 3 w/o KRT** | 0.12 (0.48) | -0.27 (0.44) | -0.47 (0.66) | -0.03 (0.45) | 0.55 (1.01) |
| **Stage 1 to Death** | **0.70 (0.07)** | **0.22 (0.07)** | **-0.52 (0.11)** | **0.34 (0.07)** | **0.27 (0.07)** | **Stage 3 w/ KRT to Death** | **0.26 (0.10)** | -0.02 (0.10) | -0.19 (0.12) | **0.29 (0.10)** | **-0.66 (0.13)** |
| **Stage 1 to Discharge** | -0.02 (0.02) | **-0.10 (0.02)** | -0.01 (0.02) | **-0.38 (0.02)** | **-1.29 (0.02)** | **Stage 3 w/ KRT to Discharge** | 0.15 (0.14) | 0.02 (0.14) | 0.12 (0.16) | **-0.26 (0.13)** | **-1.56 (0.14)** |
| **Stage 2 to No AKI** | **-0.33 (0.04)** | -0.03 (0.04) | 0.04 (0.05) | **-0.35 (0.04)** | -0.03 (0.04) |  |  |  |  |  |  |

Abbreviations. AKI, acute kidney injury; CCI, Charlson comorbidity index; ICU, intensive care unit; KRT, kidney replacement therapy. Covariates with p-value ≤ 0.05 are shown in bold face.
